# Genome and GWAS analyses for soybean cyst nematode resistance in USDA world-wide common bean (*Phaseolus vulgaris*) germplasm

**DOI:** 10.3389/fpls.2025.1520087

**Published:** 2025-03-21

**Authors:** Ainong Shi, Haizheng Xiong, Thomas E. Michaels, Senyu Chen

**Affiliations:** ^1^ Department of Horticulture, University of Arkansas, Fayetteville, AR, United States; ^2^ Department of Horticultural Science, University of Minnesota, St Paul, MN, United States; ^3^ Southern Research and Outreach Center, University of Minnesota, Waseca, MN, United States

**Keywords:** common bean, genomic prediction (GP), genomic selection (GS), genome-wide association study (GWAS), *Heterodera glycines*, *Phaseolus vulgaris*, single nucleotide

## Abstract

Soybean cyst nematode (SCN), *Heterodera glyc*ines, has become a significant threat in common bean (*Phaseolus vulgaris*) production, particularly in regions like the upper Midwest USA. Host genetic resistance offers an effective and environmentally friendly approach to managing SCN. This study aimed to conduct a genome-wide association study (GWAS) and genomic prediction for resistance to SCN HG Types 7 (race 6), 2.5.7 (race 5), and 1.3.6.7 (race 14) using 0.7 million whole-genome resequencing-generated SNPs in 354 USDA worldwide common bean germplasm accessions. Among these, 26 lines exhibited resistance to all three HG types, with a female index (FI) of less than 10. Four QTL regions on chromosomes (Chr) 2, 3, 6, and 10 were associated with resistance to HG Type 7; four regions on Chrs 2, 6, 9, and 11 were associated with resistance to HG Type 2.5.7; and three regions on Chrs 2, 6, and 10 were associated with resistance to HG Type 1.3.6.7. Cross-prediction revealed high prediction ability (PA) of 75% (r-value) for resistance to each of the three HG types. However, low PA was observed for SCN resistance through across-population prediction between the two domestications, Mesoamerican and Andean common bean accessions. Yet, using a population of mixed Mesoamerican and Andean accessions as a training set showed a high PA to predict either sub-population. This study provides SNP markers for marker-assisted selection and high PA for genomic selection in common bean molecular breeding, enabling the selection of lines and plants with high SCN resistance. Moreover, the study observed high PA for resistance among the three HG types. Interestingly, the most highly associated SNP markers and QTL for SCN resistance varied between the two domestications, and SCN resistance is more associated with the Mesoamerican domestication than the Andean domestication. This result suggests that resistance to SCN in common bean may be related to domestication rather than co-evolution with SCN.

## Introduction

The common bean (*Phaseolus vulgaris* L.) holds a vital role as the most important edible legume crop worldwide, surpassing the combined value of other food legumes like peas and chickpeas (Jain et al., 2016). Its high nutritional content makes it a crucial protein source for billions of people globally. In 2019, global production reached 28.9 million tons (Nadeem et al., 2021). While commonly harvested as dry beans, the crop is also grown as a green vegetable in many regions, known as green beans or snap beans.

The United States (US) is one of top producers in the world for common bean (Semba et al., 2021), with commercial production across 18 states, and in 2022 U.S. was the 7th largest dry bean producing country (Global Change Data Lab, 2022). The top five producing states, North Dakota, Michigan, Minnesota, Nebraska, and Idaho are responsible for a significant share of the country’s annual yield, planting 1.5 to 1.7 million acres and harvesting between 17.7 and 37.7 million tons of dry bean seeds (Shahbandeh, 2023).

The production of dry edible beans faces significant challenges from the soybean cyst nematode (SCN), particularly in regions where both soybean and bean crops are grown. SCN, scientifically known as *Heterodera glycines* Ichinohe (Tylenchida: Heteroderidae), is the most damaging pathogen affecting soybean (*Glycine max* (L.) Merr.), causing substantial yield losses in soybean-growing regions globally. In the U.S. alone, SCN is responsible for more soybean yield losses than any other pathogen (Allen et al., 2017; Bandara et al., 2020; Koenning and Wrather, 2010). Yield losses can exceed 40%, depending on factors such as SCN population density, soil characteristics, precipitation patterns, and the use of susceptible soybean varieties (Duan et al., 2009; Koenning and Wrather, 2010).

The top dry bean-producing states in the U.S., including North Dakota, Michigan, Nebraska, and Minnesota, which collectively contribute approximately 75% of the common bean production in the country (Shi et al., 2021), are also among the top ten soybean-producing states (Tylka and Marett, 2021). These regions have witnessed widespread dissemination of SCN (Tylka and Marett, 2021). Limited reports have documented damages inflicted by SCN on common bean through both field studies and greenhouse experiments (Noel et al., 1982; Poromarto et al., 2010; Trueman et al., 2022; Yan et al., 2017). Symptoms of SCN infection on common beans are similar to those on soybean, including stunting plants, yellowing leaves, reduced root mass, and discoloration of roots. The presence of cysts on roots is a sign of SCN infestation. As common bean is a suitable host for SCN, infections can lead to significant yield losses, often without obvious above-ground symptoms, making SCN a serious threat to common bean production (Poromarto and Nelson, 2009; Poromarto et al., 2010). Microplot experiments in North Dakota showed 27% to 56% dry bean yield loss depending on dry bean genotypes, environments and SCN population densities (Poromarto et al., 2010).

The use of host resistance is the most effective strategy for managing SCN in soybean. Over half of the soybean germplasm accessions in the United States have been evaluated for resistance to one or more SCN races (https://www.ars-grin.gov/). Extensive research and breeding efforts have resulted in the development of numerous SCN-resistant soybean cultivars, which are now widely planted across U.S. soybean fields.

Despite extensive research on the genetics of SCN resistance in soybean, only a few Quantitative Trait Loci (QTLs) harbor major genes that confer SCN resistance (Bent, 2022; Concibido et al., 2004; Grant et al., 2010; Guo et al., 2006; Mitchum, 2016). Among these, the *rgh1* and *Rhg4* QTLs have been extensively studied and are widely utilized in breeding commercial soybean cultivars (Bent, 2022; Mitchum, 2016). The *rgh*1 region, located on chromosome (Chr) 18 (linkage group G) in most known sources of SCN resistance, including Peking, PI 88788, and PI 437654, harbors critical genes responsible for SCN resistance (Guo et al., 2006; Kim et al., 2010; Lee et al., 2015). Copy number variations (CNV) in the *rhg1* region affect resistance, with susceptible soybeans typically possessing one copy, while two or more copies confer resistance (Cook et al., 2012). The *Rhg4* QTL, located on chromosome 8 (linkage group A2), encodes serine hydroxymethyltransferase (SHMT), also known as *GmSNAP08* (Kandoth et al., 2017; Liu et al., 2012; Patil et al., 2019; Yu et al., 2016). There are two major types of SCN resistance: Peking-type and PI 88788-type, each governed by distinct genetic mechanisms involving combinations of CNVs in *rhg1* and *Rhg4*. Recent studies have highlighted the role of additional QTLs, such as *rhg2* on chromosome 11, in contributing to SCN resistance, particularly in combination with *rhg1-a* or *Rhg4* (Basnet et al., 2022; Bent, 2022). Further QTLs, such as those on chromosome 10 in soybean line PI 567516C, have been identified, although the genetic mechanisms involved are still poorly understood. Interestingly, despite the presence of *rhg1* in PI 567516C, it does not contribute detectable SCN resistance, suggesting other mechanisms are at play (Lian et al., 2014; Usovsky et al., 2021; Vuong et al., 2010). These findings underscore the complex genetic basis of SCN resistance in soybean and the need for further research to fully understand the mechanisms and apply them in breeding programs.

In common bean, research has demonstrated resistance to soybean cyst nematode (SCN) in certain accessions and cultivars. For instance, Smith and Young (2003) evaluated 20 common bean lines in greenhouse studies and observed that Mesoamerican genotypes displayed higher resistance to SCN compared to Andean genotypes. Similarly, in North Dakota, a total of 416 USDA core accessions of *Phaseolus vulgaris* were evaluated, and around 23% of them were highly resistant to SCN HG Type 0 (Jain et al., 2019; Poromarto et al., 2012). Wen et al. (2019) conducted a study in Illinois, evaluating 363 accessions from the same core collection and identified 16 accessions (around 4.4%) with high resistance to SCN HG Type 2.5.7. These findings provide a valuable foundation for breeding programs aimed at developing SCN-resistant common bean cultivars.

Several studies have identified genetic markers and candidate genes linked to SCN resistance in common bean, although research is less extensive compared to soybean. Jain et al. (2016) performed a transcriptome analysis comparing the SCN-resistant line PI 533561 and the susceptible line GTS-900, identifying differentially expressed genes involved in plant defense. These included genes encoding nucleotide-binding site leucine-rich repeat (NLR) proteins, WRKY transcription factors, pathogenesis-related (PR) proteins, and heat shock proteins, providing key molecular insights into SCN resistance. Additionally, Wen et al. (2019) conducted genome-wide association studies (GWAS) and discovered SNP markers on chromosome 1 associated with resistance to SCN HG Type 2.5.7, near a gene cluster orthologous to the *rhg1* locus in soybean. Other resistance-related QTLs were found on chromosome 7. In further GWAS research, Jain et al. (2019) identified several QTLs related to resistance to SCN HG Type 0, spanning chromosomes 7, 8, 9, and 11. These findings provide a roadmap for understanding the genetic architecture of SCN resistance in common bean and facilitate marker-assisted selection (MAS) for breeding resistant cultivars.

A significant research project funded by the Minnesota Department of Agriculture (July 2017 to June 2020) evaluated common bean accessions for SCN resistance. Out of 315 USDA core accessions tested, 20 lines (~4.7%) exhibited resistance to SCN HG Type 0, with a female index (FI) ranging from 4.8 to 9.9, indicating reduced reproduction compared to susceptible soybean varieties (Shi et al., 2021). Subsequent GWAS analysis using the BARCBean6K_3 Infinium BeadChips identified 11 SNP markers strongly associated with resistance to SCN HG Type 0, distributed across chromosomes 4, 6, 7, 9, and 11. Further GWAS analysis extended to SCN resistance to HG Types 2.5.7 and 1.3.6.7, utilizing phenotypic data from Wen et al. (2019) and the same genotyping platform. This led to the identification of six SNP markers for HG Type 2.5.7 on chromosomes 1, 2, 3, and 7, and 12 SNP markers for HG Type 1.3.6.7 on chromosomes 1, 3, 6, 7, 9, 10, and 11 (Shi et al., 2021).

To further advance the identification of SCN-resistant lines, the screening initiative was expanded to include a broader collection of common bean germplasm from the USDA. An additional 840 lines were selected for preliminary screening, revealing significant variation in SCN resistance. Based on these findings and the core line evaluation by Shi et al. (2021), a panel of 354 purified lines was curated for further assessment of resistance to SCN HG Types 7, 2.5.7, and 1.3.6.7. This panel includes the 23 accessions with FI < 10 for HG Type 0 resistance, as identified by Shi et al. (2021). The objectives of this research are twofold: to identify additional SCN-resistant common bean germplasm and to explore the genetic mechanisms underpinning SCN resistance. These efforts aim to contribute to the development of resilient and productive common bean varieties, promoting food security and sustainability in bean production systems.

## Materials and methods

### Plant and nematode materials

A total of 354 common bean germplasm accessions were used in this study, sourced from the USDA GRIN collection. These accessions were collected from 46 countries, with a predominant focus on 10 countries, contributing 254 accessions (72.0% of the total). The major contributors were Mexico (62 accessions), Bulgaria (39), China (39), the United States (30), Turkey (20), India (17), Macedonia (14), Hungary (13), France (11), and the Netherlands (10) (**Supplementary Table S1**).

In addition, we included seven soybean SCN HG Type indicator lines: PI 548402 (Peking), PI 88788, PI 90763, PI 437654, PI 209332, PI 89772, and PI 548316 (Niblack et al., 2002), along with four SCN race differential lines: PI 548402 (Peking), PI 548982 (Pickett 71) or PI 548988 (Pickett), PI 88788, and PI 90763 (Riggs and Schmitt, 1988). Williams 82 (PI 518671) was included as a susceptible control. These lines were utilized to validate the virulence phenotypes of the SCN populations (**Supplementary Table S2**).

The 354 common bean accessions were evaluated for resistance against three SCN HG Types: 7 (race 6), 2.5.7 (race 5), and 1.3.6.7 (race 14) (**Supplementary Table S2**). These SCN populations were originally collected from fields in Swift County (2007), Waseca County (2007), and Murray County (1997), respectively, in Minnesota, USA. HG Type 7 was initially prevalent in Minnesota and the north central region, and it is avirulent to the major sources of SCN resistance soybean PI 88788 and Peking. With the use of SCN-resistant soybean cultivars for decades, most of SCN populations in the region have changed to HG Type 2.5.7 that can overcome the PI 88788 resistance. The frequency of occurrence of HG Type 1.3.6.7, which can overcome resistance from Peking, also has been increasing (Chen et al., 2010; Howland et al., 2018). Consequently, we chose these three HG Types for this study to capture a broad spectrum of resistance in common bean and identify genomic regions associated with resistance across different virulence profiles.

### SCN resistance phenotyping

Since their collection, the SCN populations were maintained either in a greenhouse on susceptible soybean cultivars or stored at -20°C. Prior to the experiment, the nematode populations were cultured on the susceptible soybean cultivar ‘Sturdy’ for approximately 45 days. Inoculum eggs were prepared using the method described by Shi et al. (2021). The experiments were carried out in a growth room (**Supplementary Figure S1**), following a randomized complete block design (RCBD) with three replicates, using the same approach as previously described by Shi et al. (2021). Briefly, each replicate consisted of two common bean plants grown in two separate cone-tainers. Additionally, control soybean plants of ‘Williams 82’ were included in each replicate, with five plants in five separate cone-tainers. The cone-tainers were filled with autoclaved soil, which comprised 80% sand and 20% field clay loam soil. Subsequently, 4,000 SCN eggs were added to each cone-tainer, and one common bean or soybean seed was sown in each cone-tainer. The cone-tainers were arranged on a rack and maintained in the growth room for 35 days with the temperature set at 28°C and daily artificial lights of 16 h. Adequate soil moisture was maintained by applying water using a sprinkler irrigation system (**Supplementary Figure S1**). No fertilizer or pesticide was applied.

The cysts (females) developed on each plant were extracted and counted following the established procedures (Shi et al., 2021). To standardize the data across different tests, Female Index (FI), rather than cyst counts were used. Female Index for each plant was determined by comparing the number of SCN females on a line to the average number of females on five Williams 82 plants, using the formula: FI = (Number of females on a given plant) × 100/(Mean number of females on Williams 82) (Riggs and Schmitt, 1988). For this calculation, the FI for Williams 82 was set to 100.

### Phenotypic data analysis

Phenotypic FI data were analyzed using analysis of variance (ANOVA) via the GLM procedure in JMP Genomics 7 (SAS Institute, Cary, NC). Descriptive statistics, including the mean, range, standard deviation (SD), standard error (SE), and coefficient of variation (CV) for FI, were calculated using the ‘Tabulate’ function. Pearson’s correlation coefficients (r) were computed to assess relationships between FI values for different SCN HG types, and the distribution of FI values was visualized using the ‘Distribution’ function in JMP Genomics 9.

Broad-sense heritability (H²) was estimated using the formula described by Holland et al. (2003) and Shi et al. (2021), where H² = σ²g/[σ²g + (σ²e/b)]. Here, σ²g represents the genetic variance, σ²e represents the residual variance, and *b* denotes the number of replicates. The estimates for σ²g and σ²e were calculated as [EMS(G) - Var(Residual)]/*b* and Var(Residual), respectively, based on values derived from the ANOVA table.

### Genotyping

DNA was extracted from fresh bean leaves using the CTAB method, and the genomic DNA was randomly sheared into fragments of approximately 350 bp. Library construction was performed using the NEBNext^®^ DNA Library Prep Kit according to the manufacturer’s instructions (Novogene, http://en.novogene.com/). The process included end repair, dA-tailing, ligation with NEBNext adapters, and PCR enrichment with P5 and indexed P7 oligos to obtain fragments between 300–500 bp. Purification and quality checks were conducted using a Qubit^®^ 2.0 fluorometer for library concentration and the Agilent^®^ 2100 bioanalyzer for insert size assessment. Quantitative real-time PCR (qPCR) was then used to confirm the effective concentration of each library. Libraries with insert sizes and effective concentrations above 2 nM were deemed suitable for Illumina^®^ high-throughput sequencing.

Qualified DNA libraries were pooled based on effective concentrations and expected data output. Paired-end sequencing (PE150 bp reads) was performed on the Illumina^®^ platform. The common bean genome reference Pvulgaris 442_v2.1, from the Phytozome website (https://genome.jgi.doe.gov/portal/pages/dynamicOrganismDownload.jsf?organism=Pvulgaris), was used for mapping short reads with the Burrows–Wheeler aligner software (BWA, 0.7.8-r455). BAM files were sorted and duplicate reads removed using SAMtools (0.1.19-44428cd), while Picard (v.1.111) was employed to merge BAM files for each sample. SNP and InDel detection and filtering were performed using GATK software (v.3.5), with annotation carried out via ANNOVAR.

A total of 24.4 million SNPs were identified across the 354 accessions on 11 chromosomes, ranging from 1.47 million SNPs on Chr 6 to 2.93 million SNPs on Chr 8. After applying filtering criteria—minor allele frequency >2%, missing allele rate (MAF) <10%, and heterozygosity rate <30%—0.7 million SNPs from whole-genome resequencing (WGR) were selected for further analyses in this study.

### Genetic diversity and population structure analysis

A model-based clustering method implemented in the STRUCTURE 2.3.4 program (Pritchard et al., 2000) was employed to infer the population structure of the 354 common bean accessions based on 6,600 SNPs, with 600 SNPs randomly selected from each of the 11 common bean chromosomes. The burn-in period was set at 50,000 iterations, followed by 10,000 Markov Chain Monte Carlo iterations, utilizing an admixture model with correlated allele frequencies independent for each run (Lv et al., 2012). Ten runs were performed for each simulated value of K, ranging from 1 to 10. The statistical value delta K was calculated for each simulated K using the formula described by Evanno et al. (2005) to identify the optimal K capturing the major structure in the data. The optimal K was determined using Structure Harvester (Earl and Vonholdt, 2012) (http://taylor0.biology.ucla.edu/structureHarvester/, accessed in 2022 but now this site was closed on September 22, 2024). Subsequently, each common bean genotype was assigned to a cluster (Q) based on the probability determined by the software that the genotype belonged in the cluster, with a cut-off probability for assignment set at 0.50 or above. Finally, a bar plot with ‘Sort by Q’ was generated to visualize the population structure among the common bean genotypes (accessions) based on the optimum K.

Genetic diversity was further assessed, and phylogenetic trees were constructed using the 6,600 SNPs in MEGA 7 (Kumar et al., 2016) based on the Maximum Likelihood tree method with specific parameters as described previously (Shi et al., 2016, 2017).

### Association analysis

GWAS were conducted following a two-step approach, as described by Shi et al. (2022) for spinach. In the first step, the BLINK (Bayesian-information and Linkage-disequilibrium Iteratively Nested Keyway) method was applied to a panel of 354 common bean accessions using 0.7 million SNPs. GWAS was performed separately for each chromosome, using phenotypic data from three SCN Female Index (FI) values: HG 2.5.7, HG 7, and HG1.3.6.7. BLINK identified 1,987 SNPs with a logarithm of odds (LOD) score [Here, we defined LOD = -log(P-value)] greater than 4.0, which were associated with resistance to one or more HG types.

In the second step, a set of 87,176 SNPs was used for GWAS, comprising the 1,987 associated SNPs from the first step and 85,367 additional randomly selected SNPs for PCA and kinship analysis. This step employed several models, including BLINK, fixed and random model circulating probability unification (FarmCPU), mixed linear model (MLM), and multiple-locus MLM (MLMM), using GAPIT 3. A *t*-test was also performed for all 87,176 SNPs in the panel of 354 accessions using Visual Basic in Microsoft Excel 2020.

The 354 accessions were divided into two sub-populations, Q1 and Q2, based on SNP data from GAPIT 3 (87,176 SNPs) or STRUCTURE 2 (6,600 SNPs). Q1 consisted of 202 accessions associated with Mesoamerican domestication, while Q2 comprised 152 accessions linked to Andean domestication. GWAS was then conducted separately for each sub-population using the four models, with 71,972 SNPs used for Q1 and 55,933 SNPs for Q2 after additional filtering.

Multiple GAPIT models were utilized to identify robust and consistent SNP markers associated with resistance to SCN HG Type 7, HG Type 2.5.7, and HG Type 1.3.6.7 in common bean. The significance threshold for associations was determined using Bonferroni correction of *P*-values with α = 0.05 (0.05/SNP number). LOD values of 6.24, 6.16, and 6.05 were used as significance thresholds for the full panel of 354 accessions, Q1, and Q2, respectively.

PCA and genetic diversity were also assessed using GAPIT 3, with PCA ranging from 2 to 10 components, and the neighbor-joining (NJ) method used to construct phylogenetic trees. NJ trees were generated for the entire panel of 354 accessions, Q1, and Q2, respectively.

### Candidate gene prediction

Candidate genes associated with SCN resistance were identified within a 50 kb region flanking both sides of the significant SNPs, following the methodology described by Zhang et al. (2016a). These candidate genes were extracted from the reference annotation of the common bean genome, using the Pvulgaris 442_v2.1 assembly, which is available through the Phytozome website (https://genome.jgi.doe.gov/portal/pages/dynamicOrganismDownload.jsf?organism=Pvulgaris).

### Genomic prediction for genomic selection of SCN resistance

In this study, ridge regression best linear unbiased prediction (RR-BLUP) was employed to predict genomic estimated breeding values (GEBV) in genomic prediction (GP). The analysis was conducted using the rrBLUP package (Endelman, 2011) in R software (Version 4.3.1, https://www.r-project.org/). RR-BLUP is widely regarded as a robust and accurate prediction method, with successful applications across a variety of crops and traits (Heslot et al., 2012; Jarquin et al., 2014; Zhang et al., 2016b). In addition, GP was performed using Bayesian models such as Bayes A (BA), Bayes B (BB), Bayes LASSO (BL), and Bayesian ridge regression (BRR), all implemented in the BGLR package. GEBV prediction was also carried out using genomic best linear unbiased prediction (gBLUP) and composite best linear unbiased prediction (cBLUP) in the GAPIT package. These approaches have been documented for their effectiveness in genomic selection (GS) in prior studies.

GP for SCN resistance was conducted across multiple panels and scenarios. Initially, GP was performed using 10 different randomly selected SNP sets, ranging from 20 to 10,000 SNPs, and two GWAS-derived SNP marker sets (20 and 71 markers, referred to as m20 and m71) for resistance to SCN HG Type 7 across three panels: the full set of 354 common bean accessions, Q1 (202 accessions), and Q2 (152 accessions). These predictions were evaluated using seven GP models (BA, BB, BL, BRR, cBLUP, gBLUP, and rrBLUP).

Next, GP was performed across nine folds (2-fold through 10-fold, with training and testing sets in different ratios) for resistance to three SCN HG types in the three panels, utilizing the rrBLUP model. GP was also conducted using nine SNP number sets (from 20 to 10,000 SNPs) in across-population prediction, comparing predictions from Q1 to Q2 or vice versa for resistance to the three SCN HG types.

Furthermore, GP was conducted with 11 combinations of across- and cross-population scenarios using all 87,176 SNPs or 10,000 SNPs in across-population prediction for resistance in the three panels (all 354 accessions, Q1, and Q2) across four GP models (maBLUP, cBLUP, gBLUP, and sBLUP) in GAPIT 3. Additionally, GP was carried out within the same SCN HG type or across different types using various SNP sets (ranging from 500 to 87,176 SNPs) across the four GP models in GAPIT3.

The correlation coefficient (r-value) was estimated among prediction values for SCN HG Types 7 (HG 7; race 6), 2.5.7 (HG 2.5.7; race 5), and 1.3.6.7 (HG 1.3.6.7; race 14) using different SNP sets. Lastly, genomic heritability (GH) was calculated for SCN resistance across the three panels using 10 randomly selected SNP sets (ranging from 20 to 10,000 SNPs) and the two GWAS-derived SNP sets (m20 and m71), estimated using rrBLUP.

The prediction accuracy of GS for SCN resistance was evaluated using the average Pearson’s correlation coefficient (r) between the GEBVs and the observed values in the validation sets. These sets were randomly generated 100 times, with the r value calculated for each iteration. The average r value across the iterations was then used to determine prediction accuracy, where higher r values indicated greater accuracy and efficiency in GS, reinforcing the reliability of GP for SCN resistance.

## Results

### SCN resistance evaluation

The reactions of common bean indicator lines (differential lines) to the soybean cyst nematode (SCN) populations are summarized in **Supplementary Table S2**. The susceptible control Williams 82 consistently exhibited over 280 average SCN females per plant across all experiments conducted for each of the three SCN HG types, indicating sufficient SCN reproduction for the study. Based on SCN HG type testing using the seven SCN indicators and race testing with the four SCN indicators the three populations were confirmed to be HG Type 7, HG Type 2.5.7, and HG Type 1.3.6.7 (**Supplementary Table S2**).

**Figure 1:**
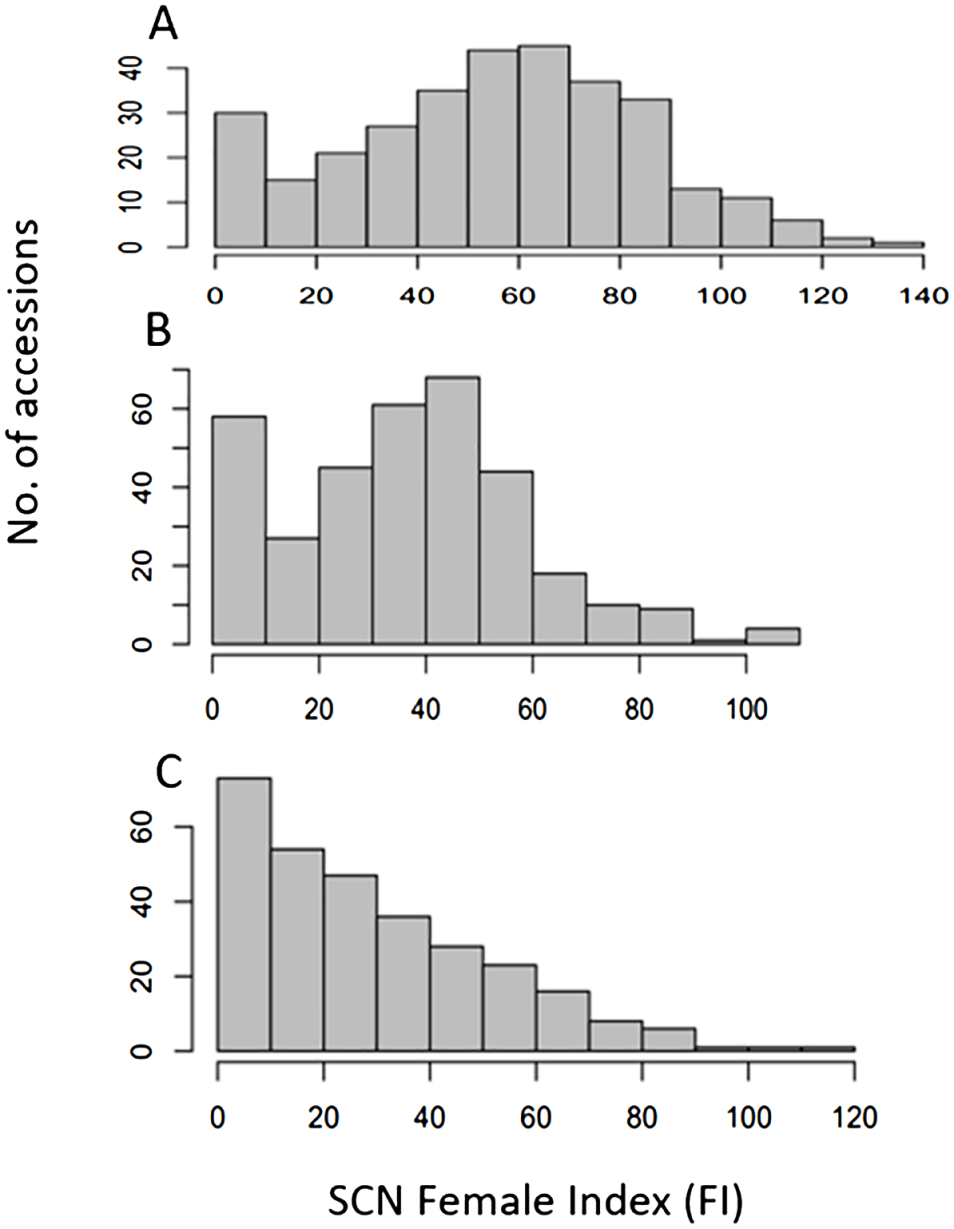
Distribution of Female Index of SCN HG Type 7 (race 6) **(A)**, HG Type 2.5.7 (race 5) **(B)**, and HG Type 1.3.6.7 (race 14) **(C)** on 354 USDA common bean accessions, where x-axis presents female index (FI) and y-axis presents number of accessions.

The Female Index (FI) values for HG Type 7 exhibited a wide range, from 1.1 for PI 417624 to 136.9 for W6 11340, with an average of 53.6, standard deviation (Std Dev) of 29.6, standard error (Std Err) of 1.6, and coefficient of variation (CV) of 52.2% (**Supplementary Tables S1**, **S3**). The distribution of FI values showed a near-normal distribution (**Figure 1**), suggesting significant variation in resistant reactions to SCN HG Type 7. Notably, 33 accessions demonstrated FI values < 10, indicating high resistance to HG Type 7. Among the top nine most resistant to HG Type 7 were W6 12201, PI 583570, PI 313733, PI 325750, PI 417657, PI 417624, PI 313444, PI 313445, and PI 313524, with FI values ≤ 3, while the two most susceptible accessions were W6 11340 with an FI of 136.9 and PI 198038 with an FI of 129.3 (**Supplementary Table S1**).

The FI for HG Type 2.5.7 displayed a substantial range, spanning 108.9 from 0.9 for PI 430206 to 109.8 for PI 661865 (**Supplementary Table S3**; **Figure 1**), with an average of 36.6, Std Dev of 22.1, Std Err of 1.2, and CV of 60.6%. The distribution of FI values showed a near-normal distribution (**Figure 1**), indicating significant variation in resistant reactions to SCN HG Type 2.5.7. Notably, 57 accessions demonstrated FI values < 10, signifying their resistance to HG Type 2.5.7. Among these, eight accessions, namely PI 313709, PI 313733, PI 325750, PI 430206, PI 449410, PI 201354, PI 313444, and PI 313524, exhibited FI values < 2, indicating high resistance to HG Type 2.5.7. The two most susceptible accessions were PI 661865 with an FI of 109.8 and PI 661952 with an FI of 107.4 (**Supplementary Table S1**).

The FI for HG Type 1.3.6.7 exhibited a wide range (110.2) from 1.1 for PI 313524 to 111.3 for PI 302537, with an average of 29.1, Std Dev of 22.7, Std Err of 1.3, and CV of 77.8% (**Supplementary Tables S1**, **S3**). The distribution of FI values displayed a skew distribution (**Figure 1**), indicating significant variance in resistant reactions to HG Type 1.3.6.7. A total of 72 accessions demonstrated FI values < 10.0, signifying resistance to the HG Type 1.3.6.7. Among these, 11 accessions, namely PI 313709, PI 313733, PI 313328, PI 201354, PI 313445, PI 313470, PI 319684, PI 325614, PI 313524, and PI 608388 exhibited FI values ≤2, indicating high resistance to HG Type 1.3.6.7. The two most susceptible accessions were PI 302537 with an FI of 111.3 and PI 324688 with an FI of 101.6 (**Supplementary Table S1**). PI 355419.

The combined analysis of resistance to the three HG Types revealed that the most consistent susceptible accession was PI 661952 with a high FI > 90 (93 – 107) for all three HG Types. Conversely, eight accessions, namely PI 313733, PI 325750, PI 346960, PI 417657, PI 201354, PI313444, PI 313445, and PI 313524, displayed FI values < 5 for resistance to all three HG Types, indicating these accessions possess high and broad resistance across HG Types 0, 2.5.7, and 1.3.6.7 (**Supplementary Table S1**). Moreover, 26 common bean accessions demonstrated SCN resistance with FI values < 10.0 across all three HG Types (**Table 1**). The correlation coefficients were 0.71 between HG Type 7 and HG Type 2.5.7, 0.71 between HG Type 7 and HG Type 1.3.6.7, and 0.76 between HG Type 2.5.7 and HG Type 1.3.6.7 in the association panel of the 354 common bean accessions; indicating their common resistance to the three SCN HG Types (**Supplementary Table S4**). Additionally, the broad sense heritability was estimated to be 63.7%, 72.6%, and 84.4% for HG Type 7, HG Type 2.5.7, and HG Type 1.3.6.7, respectively (**Supplementary Table S5**), suggesting that the resistance to each of the three SCN HG Types is highly inheritable. ANOVA revealed significant variations among PI accessions for resistance to each of the three SCN HG types (P < 0.0001) (**Supplementary Table S5**), indicating variation among these common bean accessions.

**Table 1:** List of the SNP markers associated with the resistance to HG Type 2.5.7 (race 5) based on Blink, FarmCPU, MLMM, and MLM, and a *t*-test.

SNP	Chr	Pos	MAF %	LOD (-log(P))					Beneficial _allele	Unbeneficial _allele	Set
				BLINK	FarmCPU	MLMM	MLM	*t*-test			
Chr06_30044825	6	30044825	7.0	0.65	11.77	0.55	8.01	9.60	A	G	all
Chr06_30072683	6	30072683	6.8	9.15	1.86	11.42	8.60	9.16	C	T	all
Chr09_29866343	9	29866343	37.4	1.27	14.49	8.94	6.73	34.89	A	G	all
Chr09_29870288	9	29870288	37.9	9.45	0.15	0.17	6.50	35.23	C	T	all
Chr11_1206371	11	1206371	9.0	5.93	10.81	8.86	6.01	18.25	T	C	all
Chr02_26871668	2	26871668	3.0	15.16	8.93	7.85	6.15	3.07	T	A	Q1
Chr09_28924508	9	28924508	9.6	5.50	9.13	3.27	2.97	3.34	T	C	Q1
Chr06_30044825	6	30044825	14.4	10.14	7.20	6.63	5.68	6.04	A	G	Q2
Chr11_1206371	11	1206371	21.2	4.20	7.48	4.69	3.49	7.46	T	C	Q2

### Genetic diversity and population structure analysis

**Figure 2:**
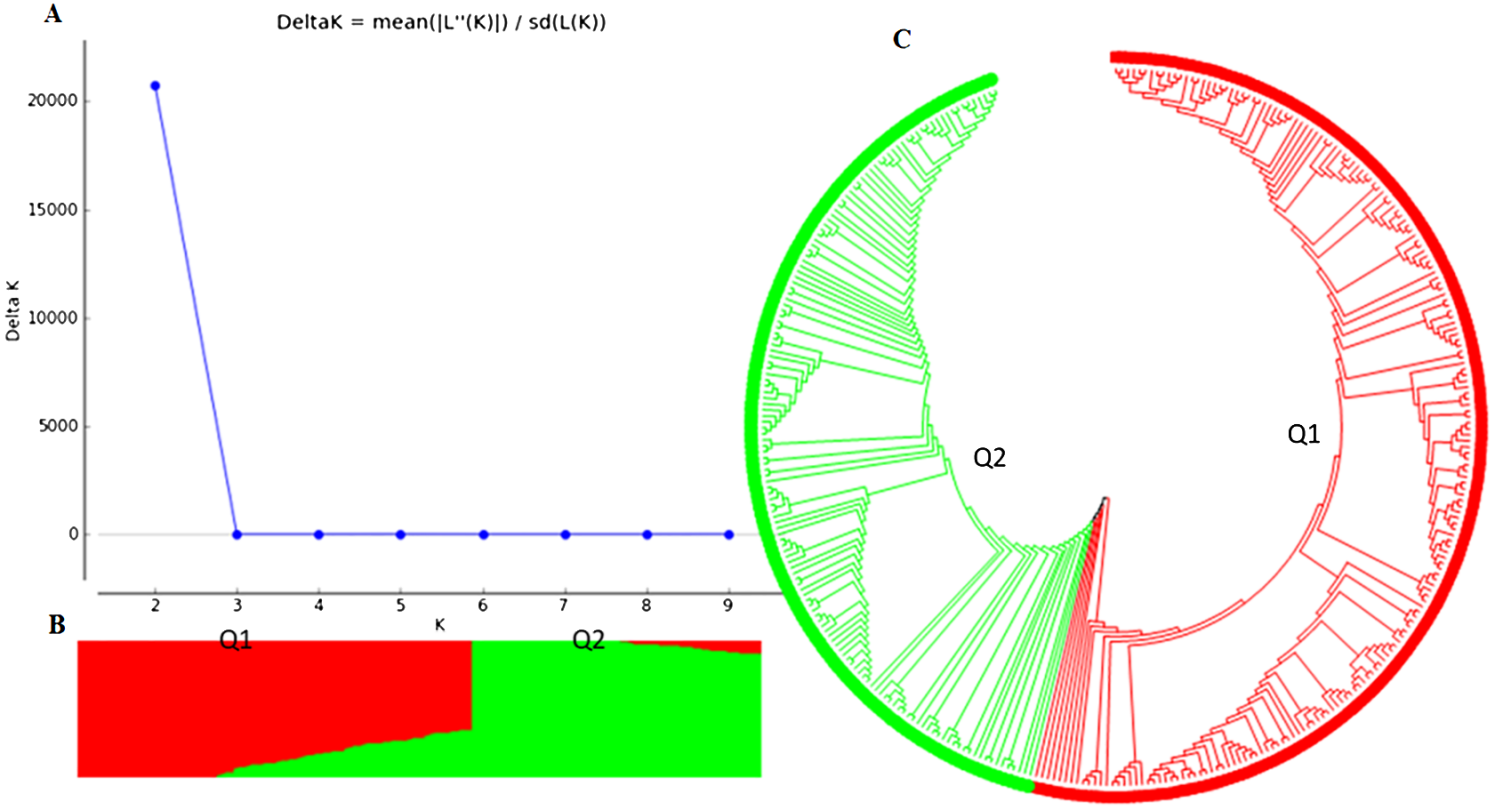
Population structure analysis of an association panel consisting of 354 USDA GRIN common bean germplasm accessions: **(A)** Delta K values for varying numbers of populations (K) inferred through analysis conducted using STRUCTURE software. **(B)** Classification of the 354 common bean accessions into two populations using STRUCTURE Version 2.3.4. **(C)** Maximum Likelihood (ML) tree depicting the genetic relationships among the 354 common bean accessions, visualized using MEGA 7.

Two main population clusters, Q1 and Q2, were observed among the 354 accessions based on STRUCTURE 2.3.4 (**Figures 2A–C**; **Supplementary Table S1**). Q1 and Q2 consisted of 202 (57.1%) and 152 (42.9%) accessions, respectively (**Supplementary Table S1**). The phylogenetic trees also showed two main clusters or populations, consistent with the STRUCTURE results, indicating at least two distinct genetic populations within the panel. The GAPIT 3 tool confirmed the presence of two sub-populations (clusters) as the best fit (**Supplementary Figures S3-2**-**S3-4** showing PCA = 2, 3, 4, and 5).

Further analysis of the Q1 sub-population (202 accessions) revealed three clusters (sub-populations) based on PCA and phylogenetic analysis when PCA = 2 to 10 in GAPIT 3, using 71,972 SNPs (**Supplementary Figure S4**). The Q2 sub-population (152 accessions) was divided into two clusters using 55,933 SNPs (**Supplementary Figure S5**).

### Association analysis

**Figure 3:**
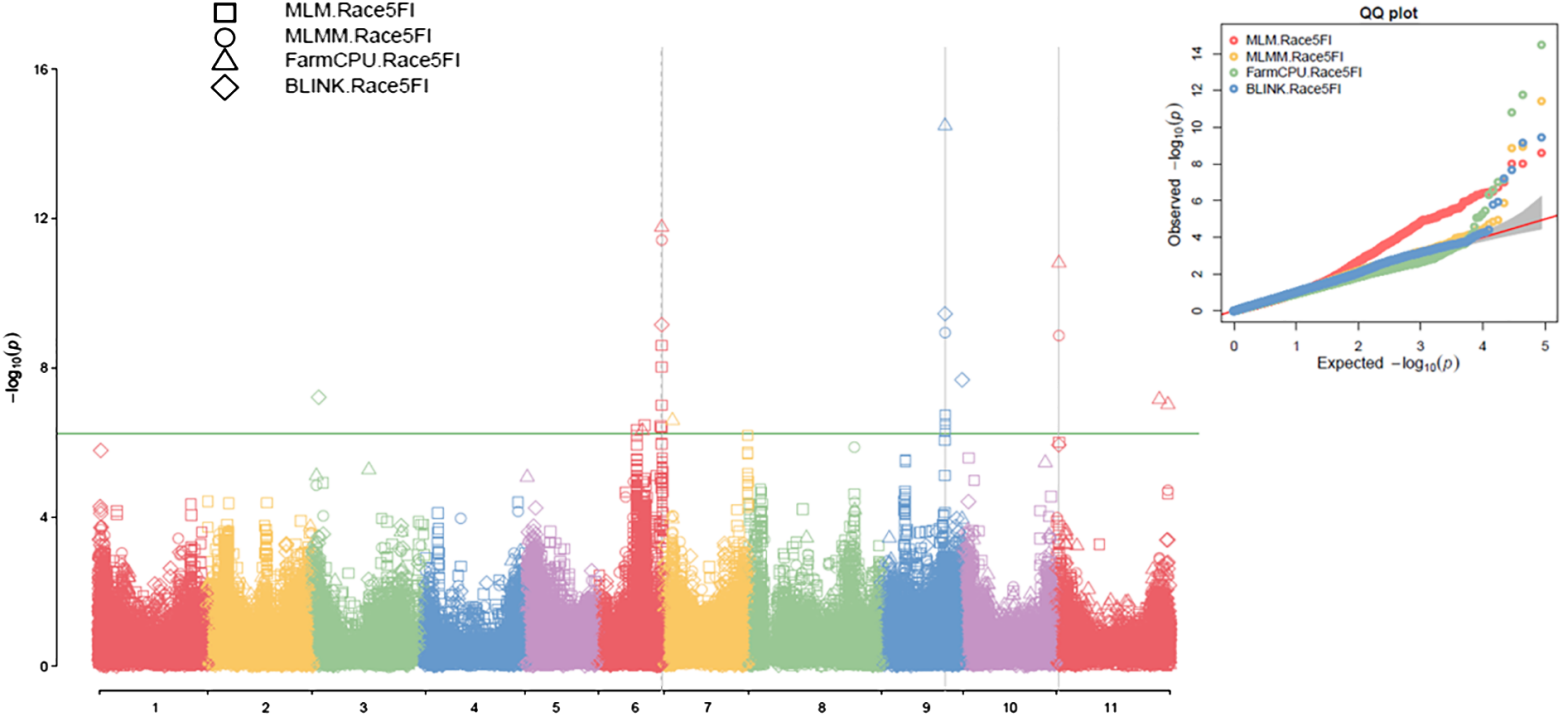
Multiple Manhattan plot (Left) and QQ plot (Right) comparing Symphysic MLM, MLMM, FarmCPU, and BLINK models in GAPIT3 for resistance to SCN HG Type 2.5.7 (Race 5) in an association panel consisting of 354 accessions: The Manhattan plot (left) illustrates common bean 11 chromosomes on the x-axis and LOD (-log(*P*-value)) values on the y-axis. The QQ plot (right) displays expected LOD (-log(*P*-value)) values on the x-axis and observed LOD (-log(*P*-value)) values on the y-axis.

**Figure 4:**
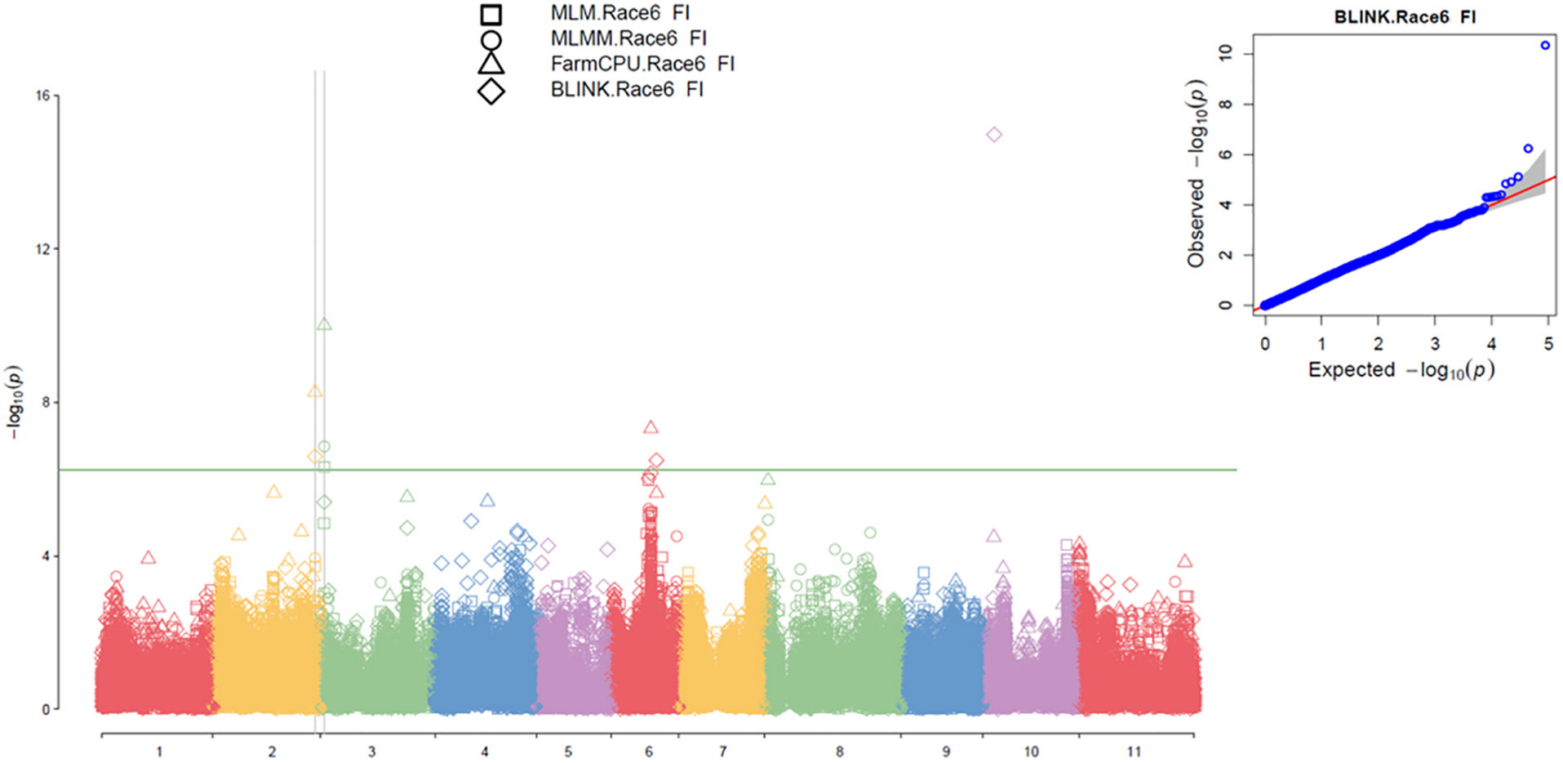
Multiple Manhattan plot (Left) and QQ plot (Right) comparing Symphysic MLM, MLMM, FarmCPU, and BLINK models in GAPIT3 for resistance to SCN HG Type 7 (Race 6) in an association panel consisting of 354 accessions: The Manhattan plot (left) displays common bean 11 chromosomes on the x-axis and LOD (-log(P-value)) values on the y-axis. The QQ plot (right) illustrates expected LOD (-log(P-value)) values on the x-axis and observed LOD (-log(P-value)) values on the y-axis.

**Figure 5:**
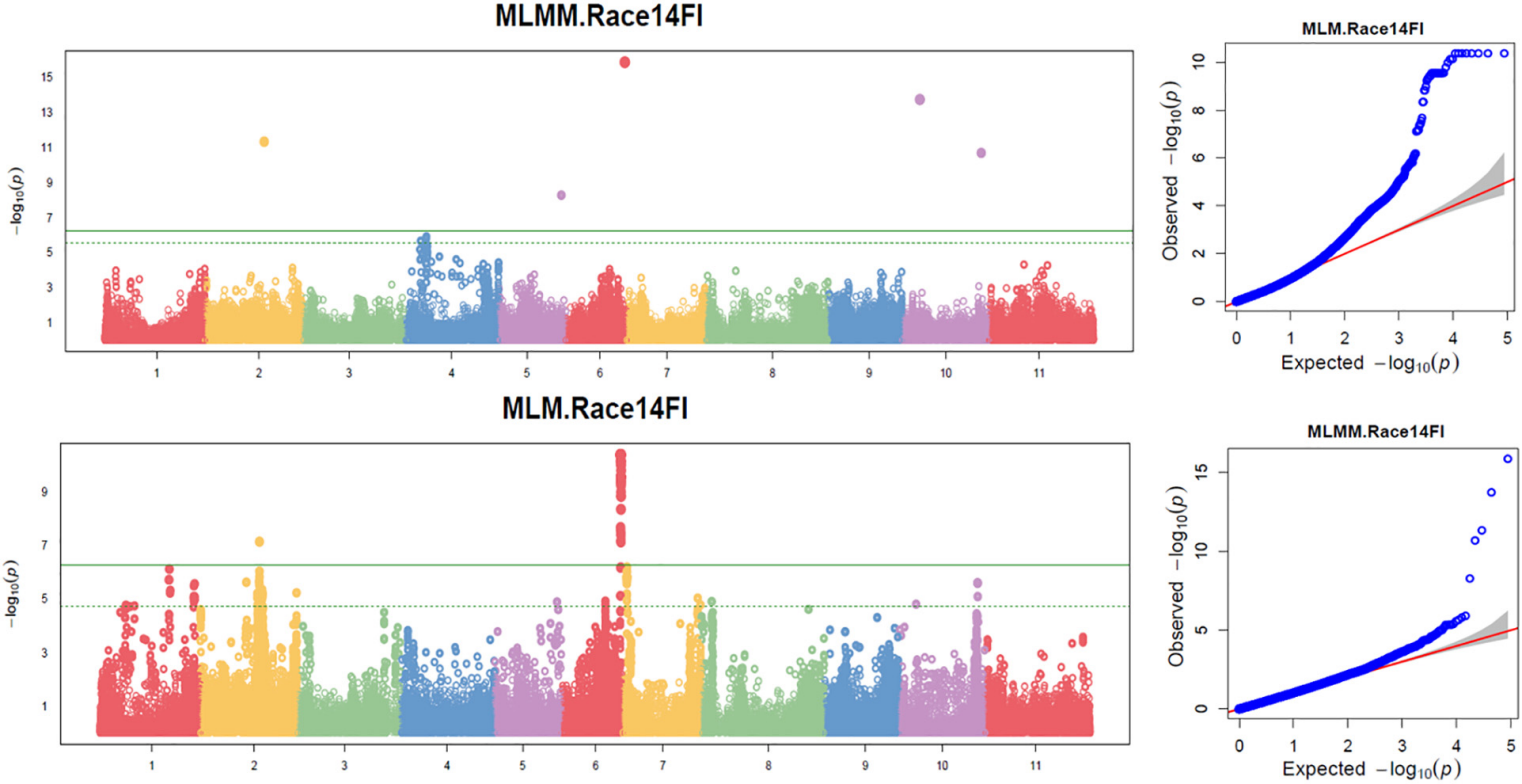
Distribution of Manhattan plots (left) and QQ-plots (right) of GWAS for resistance to SCN HG Type 1.3.6.7 (race 14) in the association panel consisted of 354 accessions based on MLMM and MLM in GAPIT3. For the Manhattan plot (Left), the x-axis presents the common bean 11 chromosomes and the y-axis for LOD (-log(P-value)) value. For the QQ-plot (right), the x-axis presents expected LOD (-log(P-value)) value and y-axis for observed LOD (-log(P-value)) value.

In this study, four GWAS models, Blink, FarmCPU, MLMM, and MLM in GAPIT 3 along with a *t*-test for each SNP were used to conduct GWAS for resistance to three HG Types (0, 2.5.7, and 1.3.6.7) across three common bean panels: all (354 accessions), Q1 (202 accessions), and Q2 (152 accessions). SNPs with a LOD (–log10(p)) value greater than 6.24 for the all panel, 6.16 for Q1, and 6.05 for Q2 from at least one of the four models for resistance to one of the three HG Types were initially listed in **Supplementary Tables S6**-**S8**, respectively, as associated SNP markers for SCN resistance. Subsequently, we selected SNPs as reliable and feasible markers if either two or more models had LOD values greater than the threshold value (6.24 in all; 6.16 in Q1; and 6.05 in Q2), or if one model had a higher LOD value or several models had LOD values close to 5. These selected SNPs were then listed in **Tables 1**–**3** for resistance to SCN HG Type 2.5.7, 7, and 1.3.6.7 (race 14), respectively. Additionally, SNP markers associated with resistance to either HG Type 7, 2.5.7, or 1.3.6.7 in two common bean panels (all plus Q1 or Q2) were compiled in **Table 4**. The multiple or single Manhattan plot and QQ plot for MLM, MLMM, FarmCPU, and BLINK models in GAPIT3 for resistance to the three SCN HG Types were visually represented in **Figures 3**–**5** for the all panel; **Supplementary Figures S6**-**S8** for Q1; and **Supplementary Figures S9**-**S11** for Q2.

**Table 2:** List of the SNP markers associated with the resistance to HG Type 7 (race 6) based on Blink, FarmCPU, MLMM, and MLM in GAPIT 3 and a *t*-test.

SNP	Chr	Pos	MAF %	LOD (-log(P))					Beneficial _allele	Unbeneficial _allele	Set
				BLINK	FarmCPU	MLMM	MLM	*t*-test			
Chr02_47299285	2	47299285	46.9	6.59	8.27	3.94	3.72	17.75	A	G	all
Chr03_1949907	3	1949907	15.4	5.40	10.01	6.85	6.31	17.91	A	G	all
Chr06_18305803	6	18305803	8.3	6.15	7.32	5.15	4.80	33.76	A	T	all
Chr10_5036799	10	5036799	21.1	14.99	1.23	2.86	2.47	48.32	A	G	all
Chr02_47299285	2	47299285	24.7	0.32	0.62	6.64	4.37	8.22	A	G	Q1
Chr02_47306325	2	47306325	24.2	11.44	8.97	0.29	4.28	9.14	G	C	Q1
Chr10_5036799	10	5036799	35.1	7.87	7.35	6.09	4.06	41.90	A	G	Q1
Chr03_1949907	3	1949907	34.6	4.89	6.22	4.89	4.39	7.48	G	A	Q2
Chr06_18293932	6	18293932	15.1	1.81	9.28	4.32	3.94	18.40	A	C	Q2

**Table 3:** List of the SNP markers associated with the resistance to HG Type 1.3.6.7 (race14) based on Blink, FarmCPU, MLMM, and MLM, and a *t*-test.

SNP	Chr	Pos	MAF %	LOD (-log(P))					Beneficial _allele	Unbeneficial _allele	Set
				BLINK	FarmCPU	MLMM	MLM	*t*-test			
Chr02_23518869	2	23518869	6.4	10.25	13.29	3.67	5.62	7.96	T	A	all
Chr06_30148782	6	30148782	9.0	0	**6.84**	0.05	**10.37**	**20.04**	T	C	all
Chr06_30220067	6	30220067	9.3	1.64	0	**15.86**	**10.37**	**20.53**	G	T	all
Chr10_39751933	10	39751933	33.9	**6.96**	0.40	0.09	3.64	**21.61**	A	T	all
Chr10_39764207	10	39764207	33.7	0.91	**7.14**	0.33	3.83	**21.53**	T	A	all
Chr02_30212013	2	30212013	37.6	**6.27**	**15.17**	**9.07**	**7.27**	2.55	G	A	Q1
Chr10_38987657	10	38987657	19.7	**7.01**	**8.11**	2.67	3.09	11.10	A	G	Q1
Chr06_30148782	6	30148782	21.2	5.73	0.09	0.29	5.24	7.81	T	C	Q2
Chr06_30220067	6	30220067	21.9	0	0.09	6.26	5.24	7.96	G	T	Q2

The bold signifies the significant LOD (-log(P)) value based on Bonferroni correction value of 6.24 in set:all; 6.16 in Q1; and 6.05 in Q2.

**Table 4:** List of seven SNP markers associated with the resistance to either HG Type 7 (race 6), 2.5.7 (race 5), or 1.3.6.7 (race14) in two common bean panels (all plus Q1 or Q2) based on Blink, FarmCPU, MLMM, and MLM, and a *t*-test.

SNP	Chr	Pos	MAF %	LOD (-log(P))					Beneficial _allele	Unbeneficial _allele	Set	Race
				BLINK	FarmCPU	MLMM	MLM	*t*-test				
Chr06_30044825	6	30044825	7.0	0.65	11.77	0.55	8.01	9.60	A	G	all	5
			14.4	10.14	7.20	6.63	5.68	6.04	A	G	Q2	
Chr11_1206371	11	1206371	9.0	5.93	10.81	8.86	6.01	18.25	T	C	all	
			21.2	4.20	7.48	4.69	3.49	7.46	T	C	Q2	
Chr02_47299285	2	47299285	46.9	6.59	8.27	3.94	3.72	17.75	A	G	all	6
			24.7	0.32	0.62	6.64	4.37	8.22	A	G	Q1	
Chr03_1949907	3	1949907	15.4	5.40	10.01	6.85	6.31	17.91	A	G	all	
			34.6	4.89	6.22	4.89	4.39	7.48	G	A	Q2	
Chr10_5036799	10	5036799	21.1	14.99	1.23	2.86	2.47	48.32	A	G	all	
			35.1	7.87	7.35	6.09	4.06	41.90	A	G	Q1	
Chr06_30148782	6	30148782	9.0	0	6.84	0.05	10.37	20.04	T	C	all	14
			21.2	5.73	0.09	0.29	5.24	7.81	T	C	Q2	
Chr06_30220067	6	30220067	9.3	1.64	0	15.86	10.37	20.53	G	T	all	
			21.9	0	0.09	6.26	5.24	7.96	G	T	Q2	

### GWAS for SCN HG Type 2.5.7 (race 5) resistance

Based on the analysis using the four models (MLM, MLMM, FarmCPU, and BLINK) in GAPIT3, the multiple QQ plot distribution showed significant deviation from the expected distribution, indicating the presence of SNPs associated with resistance to the HG Type 2.5.7 in the “all” association panel (**Figure 3** right). The multiple Manhattan plot, covering all tested 87,176 SNPs, revealed several dots (SNPs) with LOD values greater than 6.24, primarily located on Chrs 6, 9, and 11, suggesting the presence of SNPs associated with HG Type 2.5.7 resistance in the panel (**Figure 3** left). Eleven SNPs were observed with LOD values >6.24 (threshold) in one or more models for resistance to HG Type 2.5.7 in the ‘all’ panel of 354 accessions, distributed across Chrs 3, 6, 7, 9, and 11 (**Supplementary Table S6**). Among these 11 SNPs, Chr06_30044825 and Chr06_30072683, located around 30 Mb on Chr 6 spanning a length of 28 Kb, exhibited LOD values >6.24 in two and three models, respectively. Similarly, Chr09_29866343 and Chr09_29870288, located around 29.87 Mb region on Chr 9 spanning only 4 kb, showed LOD values >6.24 in three and two models, respectively, with particularly high LOD values of around 34 in the t-test. Furthermore, Chr11_1206371, positioned at 1,206,371 bp on Chr 11, demonstrated LOD values >6.24 in FarmCPU and MLMM, and approximately 6.0 in BLINK and MLM (**Table 1**). These results suggests the existence of three QTLs in the SNP regions on Chrs 6, 9, and 11 for resistance to the Type 2.5.7 in the all panel of 354 accessions.

For the Q1 panel, the QQ plots displayed a notable deviation from the expected distributions (**Supplementary Figure S6** right), indicating the presence of SNPs associated with resistance to HG Type 2.5.7. The Manhattan plots revealed multiple dots (SNPs) on Chrs 2 and 5 with LOD values exceeding 6.16 (threshold) in two or more models (**Supplementary Figure S6** left), signifying the existence of SNPs associated with the resistance to HG Type 2.5.7. Eight SNPs were identified with LOD values >6.16 in one or more models for resistance to HG Type 2.5.7 in the Q1 panel comprising 202 accessions, distributed across Chrs 1, 2, 3, 5, and 9 (**Supplementary Table S7**). Among these 8 SNPs, Chr02_26871668, positioned at 26,871,668 bp on Chr 2, exhibited LOD values >6.16 in BLINK, FarmCPU, and MLMM, and 6.15 in MLM; while Chr09_28924508, located at 28,924,508 bp on Chr 9, displayed LOD values >6.16 in FarmCPU and 5.50 in BLINK (**Table 1**), suggesting the presence of two QTLs on Chrs 2 and 9 in the regions of these two SNPs for resistance to Type 2.5.7 in the Q1 panel of 202 accessions.

For the Q2 panel, the QQ plots exhibited a noticeable deviation from the expected distributions (**Supplementary Figure S9** right half), indicating the presence of SNPs associated with resistance to HG Type 2.5.7. The Manhattan plots revealed several dots (SNPs) with LOD values exceeding 6.05 (**Supplementary Figure S9** left), indicating the existence of SNPs associated with resistance to HG Type 2.5.7. Specifically, multiple SNPs on Chr 6 displayed LOD values >6.05 in two or more models (**Supplementary Figure S9** left), suggesting the presence of stable SNP markers and QTLs for resistance to HG Type 2.5.7 on Chr 6. Five SNPs were identified with LOD values >6.05 (threshold) in one or more models for resistance to HG Type 2.5.7 in the Q2 panel distributed across Chrs 6, 9, and 11 (**Supplementary Table S8**). Among these five SNPs, Chr06_30044825, located at 30,044,825 bp on Chr 6, exhibited LOD values >6.05 in BLINK, FarmCPU, and MLMM, and 5.68 in MLM; while Chr11_1206371, located at 1,206,371 bp on Chr 11, displayed LOD values in FarmCPU and approximately 4 in the other three models (**Table 1**), suggesting the presence of a QTL on Chr 6 and 11 for resistance to HG Type 2.5.7 in the Q2 panel of 152 accessions.

The two SNP markers, Chr06_30044825 and Chr11_1206371, were identified as potential molecular markers associated with resistance to SCN HG Type 2.5.7 in both the all panel of 354 accessions and the Q2 panel of 152 accessions (**Table 4**). This confirmation suggests the presence of QTLs related to HG Type 2.5.7 resistance in the genomic regions on Chrs 6 and 11. These markers could serve as tools for marker-assisted selection in breeding programs aimed at improving resistance to HG Type 2.5.7 in common bean.

### GWAS for SCN HG Type 7 (race 6) resistance

Based on the analysis using the MLM, MLMM, FarmCPU, and BLINK models in GAPIT3, the QQ plot distributions indicated a significant deviation from the expected distribution (**Figure 4** right), suggesting the presence of SNPs associated with resistance to SCN HG Type 7 in the “all” association panel of 354 accessions. The multiple Manhattan plot displayed several dots (SNPs) with LOD values greater than 6.24 (**Figure 4** left), predominantly located on Chr 2, indicating the presence of SNPs associated with HG Type 7 resistance in the panel.

Six SNPs were identified to have LOD scores greater than 6.24 in one or more models for resistance to HG Type 7 in the all panel of 354 accessions (**Supplementary Table S6**), distributed across Chrs 2, 3, 6, and 10. Notably, Chr02_47299285, located at 47,299,285 bp on Chr 2, exhibited LOD scores exceeding 6.24 in Blink and FarmCPU models. Chr03_1949907, located at 1,949,907 bp on Chr 3, displayed LOD scores exceeding 6.24 in FarmCPU, MLMM, and MLM. Chr06_18305803, positioned at 18,305,803 bp on Chr 6, showed LOD scores greater than 6.24 in FarmCPU and over 4.5 in Blink, MLMM, and MLM. Furthermore, Chr10_5036799, located at 5,036,799 bp on Chr 10, exhibited notably high LOD scores of 14.99 in Blink and 48.32 in the t-test (**Table 2**). These SNPs were selected as markers strongly associated with resistance to HG Type 7, indicating the presence of four potential QTLs in the respective SNP regions for resistance to HG Type 7 in the all panel of 354 accessions.

For the Q1 panel, the QQ plot distributions indicated a significant deviation from the expected distribution (**Supplementary Figure S7** right), suggesting the presence of SNPs associated with resistance to SCN HG Type 7. The multiple Manhattan plot displayed several dots (SNPs) with LOD values greater than 6.16 (threshold) (**Supplementary Figure S7** left), indicating the presence of SNPs associated with SCN race 6 resistance. Notably, SNPs on Chrs 2 and 10 exhibited LOD scores greater than 6.16 in two or more models (**Supplementary Figure S7** left), suggesting the presence of stable SNP markers and QTL for resistance to SCN HG Type 7 on Chrs 2 and 10.

Six SNPs were identified to have LOD scores greater than 6.16 in one or more models for resistance to SCN HG Type 7 in the Q1 panel of 202 accessions (**Supplementary Table S7**), distributed across Chrs 2, 4, 10, and 11. Among these SNPs, Chr02_47299285, located at 47,299,285 bp on Chr 2, exhibited LOD scores exceeding 6.16 in MLMM in Q1 and was also selected as a marker in the all panel. Chr02_47306325, located at 47,306,325 bp on Chr 2, displayed LOD scores greater than 6.16 in Blink and FarmCPU models. Chr10_5036799, positioned at 5,036,799 bp on Chr 10, exhibited LOD scores exceeding 6.16 in Blink and FarmCPU and over 4.0 in MLMM and MLM. Furthermore, Chr10_5036799 had a notably high LOD value of 41.90 in the t-test. These findings suggest the presence of a QTL on Chr 2 in the region of two SNPs (Chr02_47299285 and Chr02_47306325) extending 7 Kb and another QTL in the 5 Mb region on Chr 10 for resistance to HG Type 7 in the Q1 panel of 202 accessions (**Table 2**).

For the Q2 panel, the QQ plot distributions showed a significant deviation from the expected distribution (**Supplementary Figure S10** right), indicating the presence of SNPs associated with resistance to SCN HG Type 7. The multiple Manhattan plot displayed two dots (SNPs) with LOD values greater than 6.05 on Chrs 3 and 6 (**Supplementary Figure S10** left), indicating the association of these SNPs with HG Type 7 resistance. Additionally, several SNPs on Chr 6 exhibited LOD scores greater than 6.05 in two or more models (**Supplementary Figure S10** right), suggesting the presence of stable SNP markers and QTL for resistance to HG Type 2.5.7 on Chr 6.

Two SNPs were identified to have LOD scores greater than 6.05 in one or more model for resistance to SCN HG Type 7 in the Q2 panel of 152 accessions, located on Chrs 3 and 6 (**Supplementary Table S8**). Chr03_1949907, positioned at 1,949,907 bp on Chr 3, exhibited LOD scores exceeding 6.05 in FarmCPU and over 4.3 in the four models. Chr06_18293932, located at 18,293,932 bp on Chr 6, displayed LOD scores greater than 6.05 in FarmCPU and had a notably high LOD value of 18.40 in the *t*-test (**Table 2**). These findings suggest the presence of a QTL on Chrs 3 and 6 for resistance to HG Type 7 in the Q2 panel of 152 accessions.

The three SNPs, Chr02_47299285, Chr03_1949907, and Chr10_5036799, were identified as markers for two sets: ‘all and Q1’ or ‘all and Q2’ (**Table 4**), indicating the presence of QTL in the SNP regions on Chrs 2, 3, and 10 for resistance to race 6. This suggests that these SNPs could potentially serve as reliable markers for screening SCN resistance in both the entire panel and the Q1 or Q2 subpopulations.

### GWAS for resistance to SCN HG Type 1.3.6.7 (race 14)

Based on the four models (MLM, MLMM, FarmCPU, and BLINK) in GAPIT3, the QQ plot distribution in these models between the observed vs expected LOD (-log10(p)) showed a large deviation from the expected distribution (**Figure 5**, MLMM and MLM models), indicating the presence of SNPs associated with resistance to SCN HG Type 1.3.6.7 in the “all” association panel consisting of 354 accessions. The Manhattan plots with all tested 87,176 SNPs revealed seven SNPs with LOD values greater than 6.24 (**Figure 5**), primarily located on Chrs 2, 6, and 10, indicating association with SCN resistance to the HG Type 1.3.6.7.

A total of 18 SNPs were observed to have LOD values exceeding 6.24 in one or more models for resistance to SCN HG Type 1.3.6.7 in the all panel of 354 accessions (**Supplementary Table S6**), distributed across Chrs 1, 2, 5, 6, 8, 9, and 10. Among these SNPs, Chr02_23518869 on Chr 2 exhibited LOD >6.24 in Blink and FarmCPU; one or both Chr06_30148782 and Chr06_30220067 SNPs, located on Chr 6 within an approximately 71 kb region, showed LOD >6.24 in FarmCPU, MLMM and MLM, with high LOD >20.0 in t-test; Chr10_39751933 and Chr10_39764207, situated around the 39.9 Mbp region on Chr 10, demonstrated LOD >6.24 in Blink and FarmCPU, respectively, with both showing high LOD >21.5 in t-test (**Table 3**). These SNPs were identified as markers strongly associated with resistance to HG Type 1.3.6.7, suggesting the presence of four QTLs in the SNP regions for resistance to HG Type 1.3.6.7 in the entire panel of 354 accessions.

For the Q1 panel, the QQ plot distributions between the observed and expected LOD values showed a significant deviation from the expected distribution (**Supplementary Figure S8** right), indicating the presence of SNPs associated with resistance to SCN HG Type 1.3.6.7. In the multiple Manhattan plot, numerous dots (SNPs) exhibited LOD values exceeding 6.16 (threshold) (**Supplementary Figure S8** left), indicating associations with resistance to the HG Type 1.3.6.7. Notably, SNPs located on Chrs 2 and 10 showed LOD values greater than 6.16 in two or more models (**Supplementary Figure S8** left), suggesting stable SNP markers and QTL for resistance to SCN HG Type 1.3.6.7 on these chromosomes.

A total of nine SNPs were observed to have LOD values exceeding 6.16 in one or more models for resistance to SCN HG Type 1.3.6.7 in the Q1 panel of 202 accessions (**Supplementary Table S7**), distributed across Chrs 1, 2, 6, 9, and 10. Among these SNPs, Chr02_30212013, located at 30,212,013 bp on Chr 2, exhibited LOD values exceeding 6.16 in all four models, indicating a strong association. Similarly, Chr10_38987657, situated at 38,987,657 bp on Chr 10, showed LOD values exceeding 6.16 in Blink and FarmCPU models (**Table 3**). These results suggest the presence of QTL on Chrs 2 and 10 for resistance to SCN HG Type 1.3.6.7 in the Q1 panel of 202 accessions.

For the Q2 panel, the QQ plot distributions between the observed and expected LOD values showed a notable deviation from the expected distribution (**Supplementary Figure S11** right), indicating the presence of SNPs associated with resistance to SCN HG Type 1.3.6.7. In the multiple Manhattan plots, few dots (SNPs) exhibited LOD values exceeding 6.05, mainly on Chr 6 (**Supplementary Figure S11** left). However, several SNPs on Chr 6 showed LOD values exceeding 4.0 in the MLM model, indicating associations with resistance to the HG Type 1.3.6.7.

A total of seven SNPs were observed to have LOD values exceeding 6.05 in one or more models for resistance to HG Type 1.3.6.7 in the Q2 panel of 152 accessions (**Supplementary Table S8**), distributed across Chrs 2, 4, 6, 7, 8, 9, and 10. Notably, Chr06_30148782 and Chr06_30220067, located at 30,148,782 bp and 30,220,067 bp, respectively, on Chr 6, exhibited LOD values exceeding 6.24 in FarmCPU and MLM models for Chr06_30148782, as well as in MLMM and MLM models for Chr06_30220067, with both SNPs showing high LOD values exceeding 20.0 in *t*-tests (**Table 3**). These results suggest the presence of a QTL on Chr 6 for resistance to HG Type 1.3.6.7 in the Q2 panel, with both SNPs also selected as markers in the all set.

The selection of both SNPs, Chr06_30148782 and Chr06_30220067, as markers for both the all and Q2 sets (**Table 4**) further confirms the presence of QTL in the SNP region on Chr 6 for resistance to SCN HG Type 1.3.6.7. This suggests the robustness and reliability of these markers across different panels, emphasizing their potential utility in marker-assisted breeding programs aiming to enhance resistance to HG Type 1.3.6.7 in the common bean accessions.

### Candidate gene(s) for SCN resistance

There were 138 genes (**Supplementary Table S9**) existed within the 50 Kb distance on either side of significant 20 SNP markers in **Tables 1**-**3** based on the common bean genome reference Pvulgaris 442_v2.1 at Phytozome. Among the 138 genes, five were identified as disease resistance gene analogs (**Supplementary Table S10**). Phvul.002G126600 and Phvul.002G276900, which belong to the Leucine-rich repeat protein kinase family protein, were located on Chr 2 at the regions associated with HG Type 7 resistance in the Q1 panel. Additionally, Phvul.006G207000, another member of the Leucine-rich repeat protein kinase family protein, was situated on Chr 6 and linked to SNPs Chr06_30148782 and Chr06_30220067, associated with HG Type 1.3.6.7 in both the all and Q2 panels. Phvul.011G015300, identified as a P-loop containing nucleoside triphosphate hydrolases superfamily protein, was located near SNP Chr11_1206371 on Chr 11, correlated with HG Type 2.5.7 resistance in both all and Q2 panels. Finally, Phvul.011G173801, a NB-ARC domain-containing disease resistance protein on Chr 11, was linked to SNP Chr11_48336427 and associated with HG Type 7 resistance in the Q1 panel based on GWAS results.

For resistance to HG Type 2.5.7, six genes were identified: Phvul.002G126800, Phvul.006G205300, Phvul.006G205800, Phvul.009G196500, Phvul.011G015200, and Phvul.011G015300. These genes are located on Chrs 2, 6, 6, 9, 11, and 11, respectively, each within a distance of less than 5 kb from the associated SNP markers, namely Chr02_26871668, Chr06_30044825, Chr06_30072683, Chr09_29870288, and Chr11_1206371, indicating their potential involvement in HG Type 2.5.7 resistance (**Table 5**).

**Table 5:** List of 18 disease resistance genes which are located at 5 Kb distances on upstream and dowmstream of the 15 of the 20 SNP markers in **Tables 1**–**3** associated with the three SCN HG Types.

Gene	Chr	Gene_start (bp)	Gene_end (bp)	Gene-Readable-Description	SNP	Chr	Pos (bp)	From.gene. Start (bp)	From.gene. Start (bP)	Distance	SCN race	GWAS. set
Phvul.002G126800	2	26863363	26873918	RECQ helicase L2	Chr02_26871668	2	26871668	8305	-2250	on gene	race5	Q1
Phvul.006G205300	6	30043569	30044438	EXORDIUM like 2	Chr06_30044825	6	30044825	1256	387	<1kb	race5	all,Q2
Phvul.006G205800	6	30076072	30080766	methyl-CPG-binding domain 8	Chr06_30072683	6	30072683	-3389	-8083	<4kb	race5	all
Phvul.009G196500	9	29873739	29877359	Co-chaperone GrpE family protein	Chr09_29870288	9	29870288	-3451	-7071	<4kb	race5	all
Phvul.011G015200	11	1202132	1204327	tonoplast intrinsic protein 5;1	Chr11_1206371	11	1206371	4239	2044	<3Kb	race5	all,Q2
Phvul.011G015300	11	1205296	1211056	P-loop containing nucleoside triphosphate hydrolases superfamily protein						on gene		
Phvul.002G304700	2	47290820	47294656	Mitochondrial ATP synthase subunit G protein	Chr02_47299285	2	47299285	8465	4629	<5kb	race6	all, Q1
Phvul.002G304800	2	47299804	47303649	N-MYC downregulated-like 1	Chr02_47306325	2	47306325	6521	2676	<3Kb	race6	Q1
Phvul.003G020400	3	1947544	1952837	MMS ZWEI homologue 1	Chr03_1949907	3	1949907	2363	-2930	on gene	race6	all, Q2
Phvul.010G034600	10	5037704	5048093	like heterochromatin protein (LHP1)	Chr10_5036799	10	5036799	-905	-11294	<1kb	race6	all,Q1
Phvul.002G109600	2	23521662	23522544	SAUR-like auxin-responsive protein family	Chr02_23518869	2	23518869	-2793	-3675	<3kb	race14	all
Phvul.002G149500	2	30216646	30218163	DORNROSCHEN-like	Chr02_30212013	2	30212013	-4633	-6150	<5kb	race14	Q1
Phvul.006G206700	6	30144607	30149315	CD2-binding protein-related	Chr06_30148782	6	30148782	4175	-533	on gene	race14	Q2
Phvul.006G206800	6	30153391	30158718	pumilio 7				-4609	-9936	<5kb	race14	all
Phvul.006G207500	6	30214988	30216876	Bifunctional inhibitor/lipid-transfer protein/ seed storage 2S albumin superfamily protein	Chr06_30220067	6	30220067	5079	3191	<4kb	race14	all
Phvul.006G207600	6	30224677	30225373	DNAJ-like 20				-4610	-5306	<5kb	race14	Q2
Phvul.010G113600	10	38985436	38991418	UDP-Glycosyltransferase superfamily protein	Chr10_38987657	10	38987657	2221	-3761	on gene	race14	Q2
Phvul.010G118300	10	39752572	39758271	CBL-interacting protein kinase 9	Chr10_39751933	10	39751933	-639	-6338	<1kb	race14	all

For resistance to SCN HG Type 7, four genes were identified: Phvul.002G304700, Phvul.002G304800, Phvul.003G020400, and Phvul.010G034600. These genes are located on Chrs 2, 2, 3, and 10, respectively, each within a distance of 5 kb from the associated SNP markers, namely Chr02_47299285, Chr02_47306325, Chr03_1949907, and Chr10_5036799, suggesting their potential involvement in race 6 resistance (**Table 5**).

For resistance to HG Type 1.3.6.7 (race 14), eight genes were identified: Phvul.002G109600, Phvul.002G149500, Phvul.006G206700, Phvul.006G206800, Phvul.006G207500, Phvul.006G207600, Phvul.010G113600, and Phvul.010G118300. These genes are located on Chrs 2, 2, 6, 6, 6, 6, 10, and 10, respectively, each within a distance of 5 kb from the associated SNP markers, namely Chr02_23518869, Chr02_30212013, Chr06_30148782, Chr06_30220067, Chr10_38987657, and Chr10_39751933, indicating their potential involvement in race 14 resistance (**Table 5**).

### Genomic prediction for SCN resistance

#### Genomic prediction in SNP sets with different SNP numbers

GP was estimated with 12 SNP sets, including 10 different randomly selected SNP number sets and two GWAS-derived SNP marker sets, for resistance to the three HG Types across three panels, estimated by seven GP models (**Supplementary Tables S11**-**S13**, **Supplementary Figures S12a, b**, **S13a, b**, **S14a, b**).

**Figure 6:**
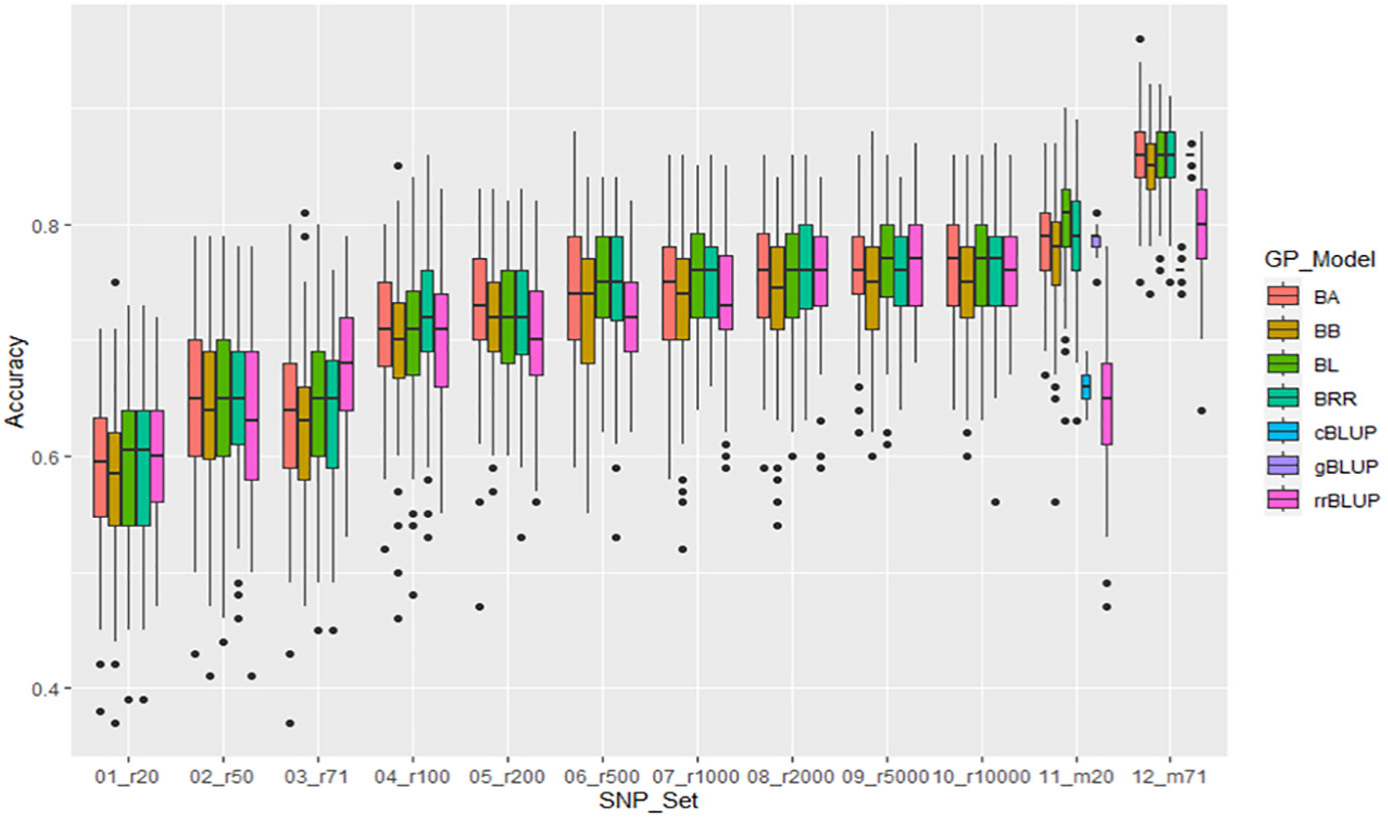
Genomic prediction (r-value) of 10 different randomly selected SNP number sets from 20 SNPs to 10,000 SNPs plus two GWAS-derived SNP marker sets (20 and 71 markers - m20 and m71) in cross-prediction for the resistance to SCN HG Type 2.5.7 (race 5) in the “all” panel of the 354 common bean accessions estimated by seven GP models (BA, BB, BL, BRR, cBLUP, gBLUP, and rrBLUP).

For resistance to HG Type 2.5.7, the mean of GP estimated by 5 models ranged from 0.59 in r20 to 0.76 in r10000 among the 10 randomly selected SNP sets. The r-value increased when more SNP numbers were used, and the two GWAS-derived SNP marker sets (m20 and m71) exhibited very high mean r-values of 0.75 and 0.83, respectively, in the all panel of 354 common bean accessions (**Supplementary Table S11**, **Figure 6**, **Supplementary Figures S12a, b**), suggesting that GP was high and resistance to HG Type 2.5.7 could be effectively selected in common bean breeding through genomic selection. Similar results were observed in the Q1 and Q2 panels, although the mean r-values were slightly lower (**Supplementary Table S11**, **Supplementary Figures S12a, b**).

**Figure 7:**
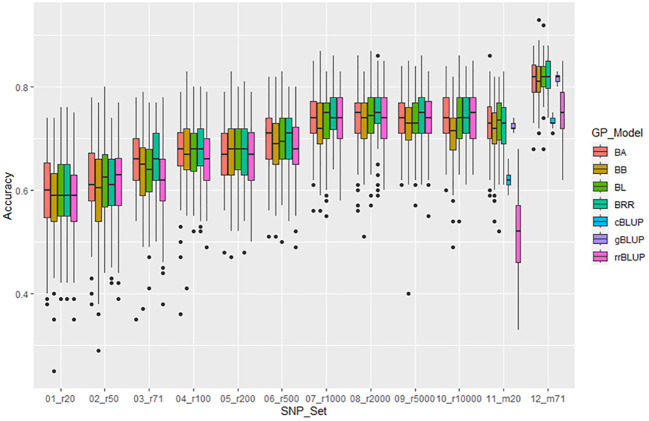
Genomic prediction (r-value) of 10 different randomly selected SNP number sets from 20 SNPs to 10,000 SNPs plus two GWAS-derived SNP marker sets (20 and 71 markers - m20 and m71) in cross-prediction for the resistance to SCN HG Type 7 (race 6) in the ‘all’ panel of the 354 common bean accessions estimated by seven GP models (BA, BB, BL, BRR, cBLUP, gBLUP, and rrBLUP).

For resistance to SCN HG Type 7, the mean of GP estimated by 5 models ranged from 0.59 in r20 to 0.74 in r1000, r2000, and r5000 among the 10 randomly selected SNP sets. The r-value increased when more SNP numbers were used from 20 to 1000 SNPs; with 1000 or more SNPs, the r-value remained similar at 0.73-0.74. The two GWAS-derived SNP marker sets (m20 and m71) exhibited very high mean r-values of 0.68 and 0.80, respectively, in the all panel of 354 common bean accessions (**Supplementary Table S12**, **Figure 7**, **Supplementary Figures S13a, b**), indicating that GP was high and resistance to HG Type 2.5.7 could be effectively selected in common bean breeding through genomic selection. Similar results were observed in Q1 with slightly higher values, but Q2 panels had slightly lower r-values (**Supplementary Table S12**, **Supplementary Figures S13a, b**).

**Figure 8:**
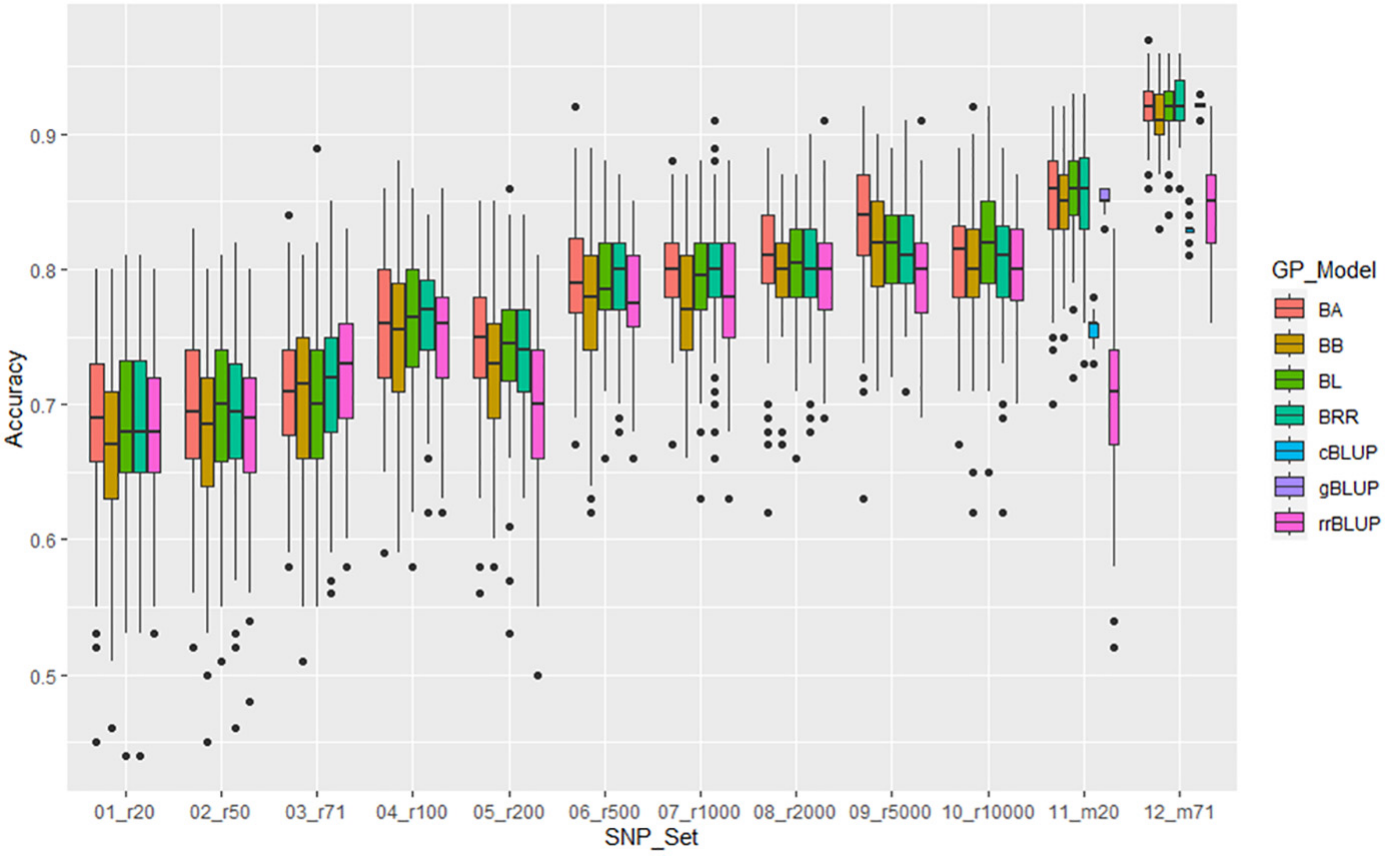
Genomic prediction (r-value) of 10 different randomly selected SNP number sets from 20 SNPso 10,000 SNPs plus two GWAS-derived SNP marker sets (20 and 71 markers - m20 and m71) in cross-prediction for the resistance to SCN HG Type 1.3.6.7 (race 14) in the ‘all’ panel of the 354 common bean accessions estimated by seven GP models (BA, BB, BL, BRR, cBLUP, gBLUP, and rrBLUP).

For resistance to SCN HG Type 1.3.6.7, the mean of genomic prediction (GP) estimated by 5 models ranged from 0.68 in r20 to 0.81 in r10000 among the 10 SNP sets randomly selected. The r-value increased as more SNP numbers were used, and the two GWAS-derived SNP marker sets (m20 and m71) exhibited high mean r-values of 0.78 and 0.90, respectively, in the all panel of 354 common bean accessions (**Supplementary Table S13**, **Figure 8**, **Supplementary Figures S14a, b**). These findings suggest that GP was high, and the resistance to HG Type 1.3.6.7 can be effectively selected in common bean breeding through genomic selection. Similar results were observed in Q1 and Q2 panels, albeit with slightly lower mean r-values (**Supplementary Table S13**, **Supplementary Figures S14a, b**).

### Genomic prediction with different folds (training and testing panel ratio)

GP (r-value) was estimated in nine folds ranging from 2-fold (training set: testing = 1:1) to 10-fold (training set: testing = 9:1) in cross-prediction for the resistance to three HG Types: HG 7, HG 2.5.7, and HG 1.3.6.7 (race 14), across three panels: all 354 accessions, Q1 with 202 accessions, and Q2 with 152 accessions, estimated by rrBLUP (**Supplementary Table S14**, **Supplementary Figure S15**). The results indicated that (1) the r-value averaged 0.75 and ranged from 0.74 in 2-fold to 0.76 in 5-, 7-, 8-, and 9-fold in the all-panel; (2) in Q1, the r-value averaged 0.76 and ranged from 0.74 in 2-fold to 0.78 in 6-fold; (3) in Q2, the r-value averaged 0.54 and ranged from 0.51 in 2-fold to 0.56 in 7- and 8-fold; (4) the r-value remained consistent across all folds from 2- to 10-fold; (5) the r-value was similar across different HG Types, averaging 0.66, 0.67, and 0.72; (6) 2-fold had the smallest r-value but also the smallest standard error (SE) value; (7) as the fold increased, the SE also increased; (8) both all and Q1 panels exhibited similar r-values around 0.75, while Q2 had a lower r-value around 0.54 (**Supplementary Table S14**, **Supplementary Figure S15**), suggesting that all nine folds can be utilized in genomic selection for SCN resistance across the three HG Types in common bean.

### Genomic prediction by across-population among common bean panels

**Figure 9:**
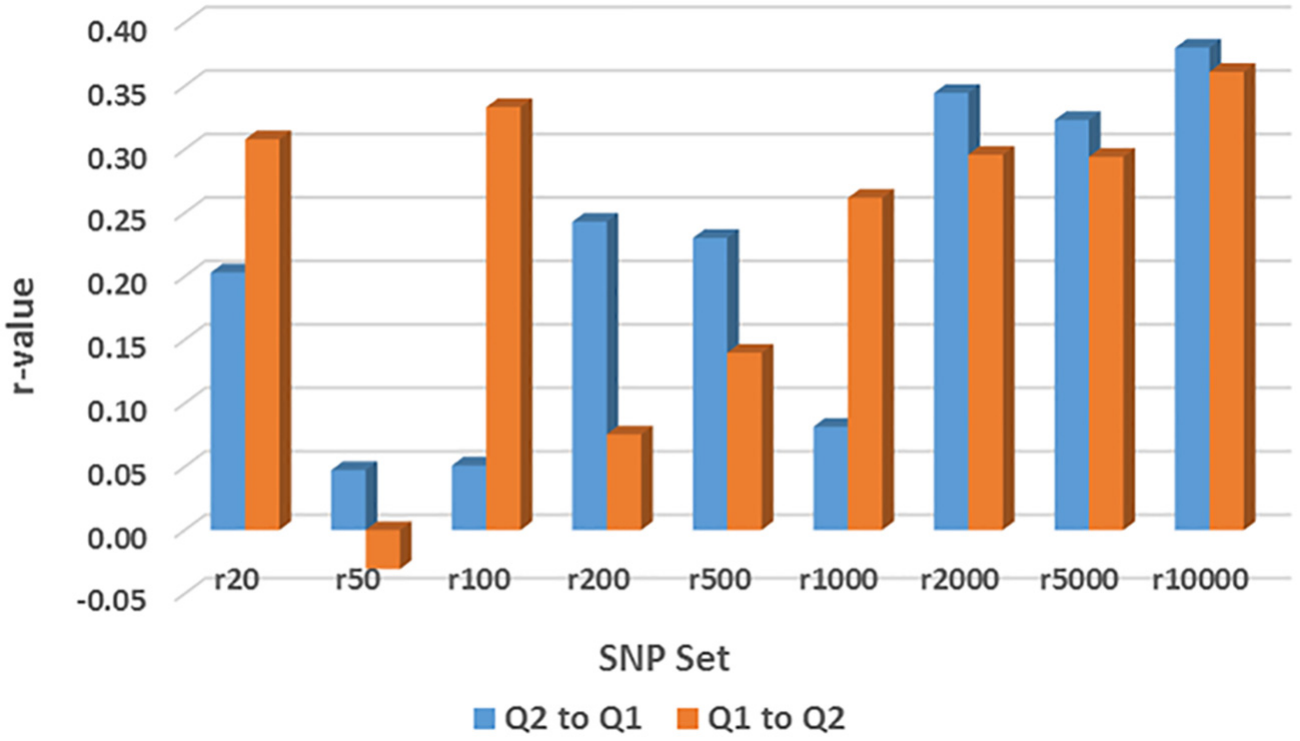
Genomic prediction (r-value) of nine different randomly selected SNP number sets from 20 SNPs to 10,000 SNPs in across-population prediction from Q1 (202 accessions) to Q2 (152 accessions) or from Q2 to Q1 for the resistance to SCN HG Type 1.3.6.7 (race 14) estimated by rrBLUP as an example.

GP (r-value) was estimated by rrBLUP across nine different randomly selected SNP number sets ranging from 20 SNPs to 10,000 SNPs in across-population prediction, either from Q1 (202 accessions) to Q2 (152 accessions) or from Q2 to Q1, for the resistance to the three HG Types (**Supplementary Table S15**; **Figure 9**). However, all GP values showed an r-value less than 0.4, indicating a low level of genetic accuracy.

For resistance to HG Type 2.5.7, all r-values in the 9 SNP sets were ≤0.11 for GP from Q1 to Q2; 8 r-values in the 9 SNP sets were ≤0.03 except for r = 0.33 in r200, and 7 were zero or below zero for GP from Q2 to Q1 (**Supplementary Table S15**). These results indicate that genomic selection will not be efficient for selecting HG Type 2.5.7 resistance through across-population prediction from Q1 to Q2 or Q2 to Q1.

For resistance to HG Type 7, the r-values were 0.27, -0.06, 0.14, 0.11, 0.01, 0.23, 0.27, 0.28, and 0.26 in r20, r50, r100, r200, r500, r1000, r2000, r5000, and r10000, respectively, showing r ≤ 0.28 in all nine SNP sets for GP from Q1 to Q2. Similarly, the r-values were 0.17, -0.10, -0.13, 0.24, -0.09, 0.10, 0.15, 0.25, and 0.15 in r20, r50, r100, r200, r500, r1000, r2000, r5000, and r10000, respectively, showing r ≤ 0.25 in all nine SNP sets for GP either from Q2 to Q1 or from Q2 to Q1 (**Supplementary Table S15**). These results indicate that genomic selection will not be highly efficient for selecting HG Type 7 resistance through across-population prediction from Q1 to Q2 or Q2 to Q1.

For resistance to HG Type 1.3.6.7, the r-values were 0.31, -0.03, 0.33, 0.08, 0.14, 0.26, 0.30, and 0.29 in r20, r50, r100, r200, r500, r1000, r2000, r5000, and r10000, respectively, showing r ≤ 0.28 in all nine SNP sets for GP from Q1 to Q2. Similarly, the r-values were 0.20, 0.05, 0.05, 0.24, 0.23, 0.08, 0.34, 0.32, and 0.38 in r20, r50, r100, r200, r500, r1000, r2000, r5000, and r10000, respectively, showing r ≤ 0.38 in all nine SNP sets for GP either from Q2 to Q1 or from Q2 to Q1 (**Supplementary Table S15**, **Figure 9**). These findings indicate that genomic selection will not be highly efficient for selecting HG Type 1.3.6.7 resistance through across-population prediction from Q1 to Q2 or Q2 to Q1. However, r ≥ 0.29 up to 0.38 were observed in r2000, r5000, and r10000 when 2000 SNPs were used, suggesting there are alleles for HG Type1.3.6.7 resistance in both sub-populations (Q1 and Q2) of the two domestic germplasm sets: Mesoamerican and Andean, and genomic selection will be less effective by across-prediction between the two sets.

### Genomic prediction by across- and cross-population among common bean panels

**Figure 10:**
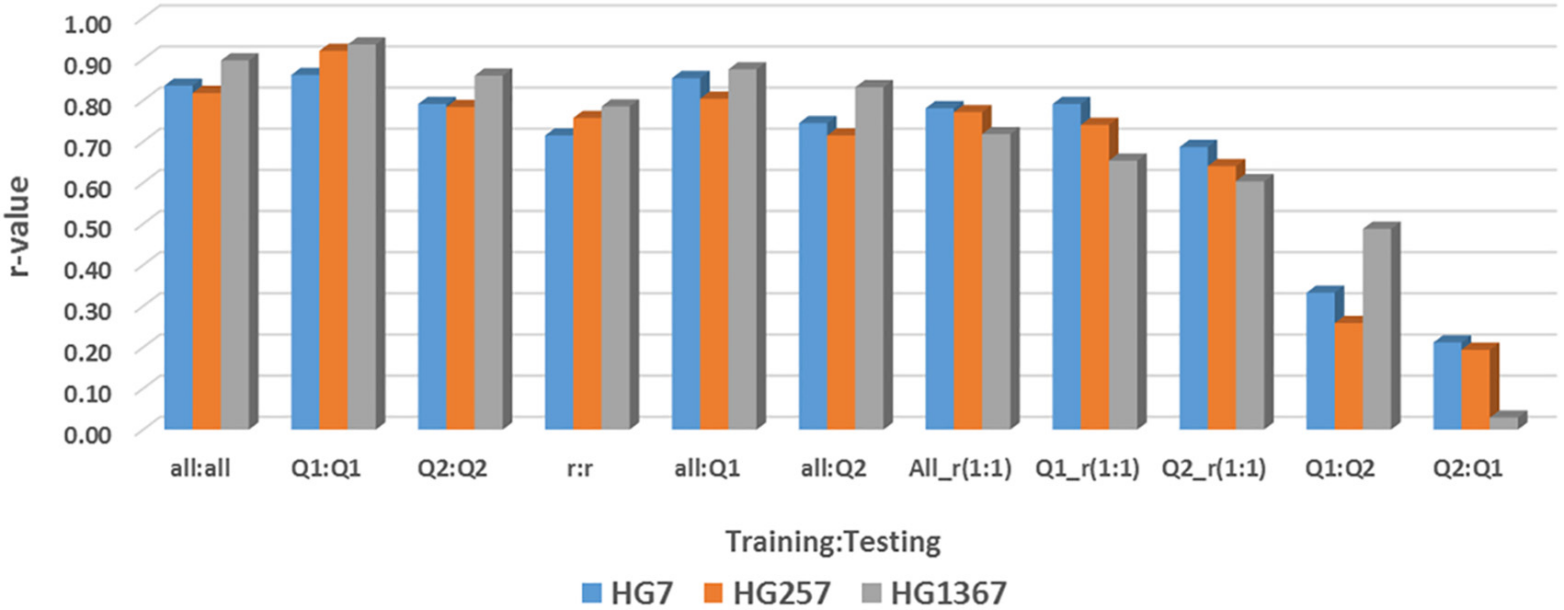
Genomic prediction (r-value) of 11 GP pairs (combinations) of across- and cross-population using 10,000 SNPs in across-population prediction all panel (354 accessions), Q1 (202 accessions), and Q2 (152 accessions) for the resistance to three SCN HG Types GH256 (race 5), GH7 (race 6), and HG 1.3.6.7 (race 14) estimated by maBLUP in GAPIT 3 as an example.

The GP (r-value) of 11 GP pairs (combinations) of across- and cross-population were estimated using all 87,176 SNPs and 10,000 SNPs as SNP sets in across-population prediction in all panel, Q1, and Q2 for the resistance to the three HG Types estimated by four GP models, maBLUP, cBLUP, gBLUP, and sBLUP in GAPIT 3 (**Supplementary Table S16**; **Figure 10**), where (1) all:all = the all 354 common bean accessions as both training and testing sets; (2) Q1:Q1 = the 202 accessions of Q1 as both training and testing sets; (3) Q2:Q2 = the 152 accessions of Q2 as both training and testing sets; (4) r:r = randomly selected 50% accessions from all 354 accessions both training and testing sets; (5) all:Q1 = the all accessions as the training set and Q1 as the testing sets; (6) all:Q2 = the all accessions as the training set and Q2 as the testing sets; (7) All_r(1:1) = randomly selected 50% accessions from all 354 accessions as the training set and the left 50% of 177 accessions as the testing sets; (8) Q1_r(1:1) randomly selected 50% accessions from the 202 accessions of Q1 as the training set and the left 50% of 101 accessions as the testing sets; (9) Q2_r(1:1) = randomly selected 50% accessions from 152 accessions of Q2 as the training set and the left 50% of 76 accessions as the testing sets; (10) Q1:Q2 = the Q1 as the training set and Q2 as the testing sets; and (11) Q2:Q1 = the Q2 as the training set and Q1 as the testing sets. The cross-population predictions within the same population include above (1) all:all, (2) Q1:Q1, (3) Q2:Q2, and (4) r:r; the cross-population predictions from a large population to a sub-population within the large population include above (5) all:Q1 and (6) all:Q2; the cross-population predictions from half sub-population to another half within the population include (7) All_r(1:1), (8) Q1_r(1:1), and (9) Q2_r(1:1); and the across-population predictions from one population to another include (10) Q1:Q2 and (11) Q2:Q1.

All cross-predictions showed high r values in both SNP sets (all_87176SNP and r1000) in each of the four GP models (maBLUP, cBLUP, gBLUP, and sBLUP) (**Supplementary Table S16**; **Figure 10** as an example for maBLUP). The mean r-value of the nine sets of training:testing, all:all, Q1:Q1, Q2:Q2, r:r, all:Q1, all:Q2, All_r(1:1), Q1_r(1:1), and Q2_r(1:1) were 0.80, 0.83, 0.82, 0.74, 0.76, 0.67, 0.72, 0.71, and 0.57, respectively, in the all_87176SNP SNP set; 0.79, 0.83, 0.74, 0.72, 0.74, 0.65, 0.71, 0.67, and 0.58, respectively, in the r1000 SNP set; and 0.79, 0.83, 0.78, 0.73, 0.75, 0.66, 0.71, 0.69, and 0.57, respectively, in the combined two SNP sets (**Supplementary Table S16**; **Figure 10**). These results indicate that the r-value was high, ≥0.67, even >0.90 in many cases, except in Q2_r(1:1) where r=0.57 averaged, suggesting that genomic selection will be efficient through cross-prediction when selection is performed within the same population (all:all, Q1:Q1, Q2:Q2, r:r), from a large population to a sub-population within the large population (all:Q1 and all:Q2), and from one half sub-population to another half within the population [All_r(1:1), Q1_r(1:1), and Q2_r(1:1)] for SCN resistance in common bean.

The mean in all cross-prediction combined was 0.75, 0.76, and 0.70 for HG 7, HG 2.5.7, and HG 1.3.6.7, respectively, in the all_87176 SNP set; and 0.78, 0.68, and 0.69 for HG 7, HG 2.5.7, and HG 1.3.6.7, respectively, in the r1000 SNP set (**Supplementary Table S16**). The r-value was high, ≥0.68, for each of the three HG Types in both SNP sets, indicating that genomic selection will be efficient to select the resistance to each of the three HG Types by cross-prediction in common bean.

The mean r-value of the two sets of training:testing, Q1:Q2 and Q2:Q1, were 0.21 and 0.15 in the all_87176 SNP set; 0.26 and 0.20 in the r1000 SNP set; and 0.24 and 0.18 in the combined two SNP sets (**Supplementary Table S16**). These values indicate that the r-value was low, ≤0.26, and even zero or below zero were observed, such as for HG 2.5.7 in the all_87176 SNP set, where r = -0.32 and -0.36 in Q2:Q1 by cBLUP and gBLUP, respectively. This suggests that genomic selection will not be efficient through across-prediction between the two common bean populations, Q1 to Q2 or Q2 to Q1, for SCN resistance.

The mean r-value of the two across-prediction combined was 0.38, 0.04, and 0.13 for HG 7, HG 2.5.7, and HG 1.3.6.7, respectively, in the all_87176 SNP set; and 0.26, 0.21, and 0.23 for HG 7, HG 2.5.7, and HG 1.3.6.7, respectively, in the r1000 SNP set (**Supplementary Table S16**). These values indicate that the r-value was low, ≥0.26, for each of the three HG Types in both SNP sets, suggesting that genomic selection will have low efficiency in selecting the resistance to each of the three HG Types through across-prediction in common bean.

The mean r-values of the four GP models were 0.70, 0.48, 0.76, and 0.65 in the all_87176 SNP set; 0.69, 0.46, 0.77, and 0.59 in the r1000 SNP set; and 0.70, 0.47, 0.76, and 0.62 in the combined two SNP sets (**Supplementary Table S16**). These results indicate that the r-values were high, ≥0.59, for each of the three HG Types in maGLUP, gBLUP, and sBLUP, but the r-value was 0.47 in cBLUP. This suggests that genomic selection will be efficient in selecting the resistance to each of the three HG Types using each of the four GP models in common bean, with gBLUP exhibiting the highest efficiency, followed by maBLUP, sBLUP, and then cBLUP.

### Genomic prediction for SCN resistance through different HG types

**Figure 11:**
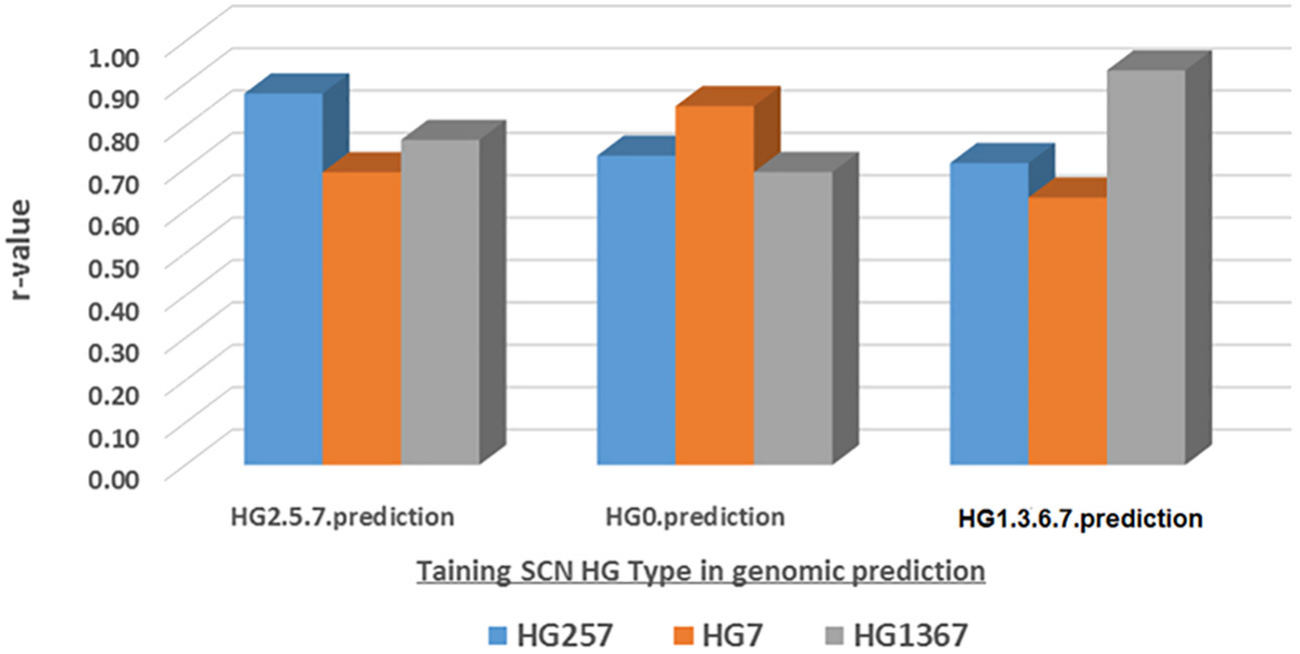
Genomic prediction (r-value) based the SCN FI in one SCN HG Type as training set to predict itself and other HG Types among the all panel (354 common bean accessions) estimated by maBLUP using all 87,176 SNPs an example for this figure.

The genomic prediction (GP) was estimated for the SCN resistance to one HG Type, either HG 7, HG 2.5.7, or HG 1.3.6.7, from another HG Type FI data using five SNP sets (all_87176, r500, r1000, r5000, and r10000) and four GP models (maBLUP, gBLUP, cBLUP, and sBLUP) (**Supplementary Table S17**; **Figure 11**, maBLUP as an example).

The mean r-values were 0.77, 0.76, and 0.75 in the maBLUP model; 0.57, 0.66, and 0.47 in the cBLUP model; 0.82, 0.81, and 0.80 in the gBLUP model; and 0.78, 0.76, and 0.67 in the sBLUP model for resistance to HG 2.5.7, HG 7, and HG 1.3.6.7, respectively (**Supplementary Table S17**). These results indicate that the r-values were high for each of the three HG Types in three models, with r-values greater than or equal to 0.67, except for cBLUP, which had r-values greater than or equal to 0.47.

The mean r-values were 0.74, 0.71, 0.72, 0.76, and 0.73 for HG 2.5.7; 0.75, 0.73, 0.75, 0.76, and 0.75 for HG 7; 0.66, 0.65, 0.66, 0.71, and 0.68 for HG 1.3.6.7; and 0.72, 0.70, 0.71, 0.74, and 0.72 for the combined set in all.87176, r500, r1000, r5000, and r10000, respectively (**Supplementary Table S17**). These results indicate that high r-values were observed, with r-values greater than or equal to 0.65. This suggests that all SNP sets had high r-values and that a set with more SNPs had slightly higher r-values, although the difference was less than 5%. Therefore, it indicates that 500 or more SNPs can be used as a SNP set in genomic selection to select SCN resistance based on phenotypic data from one HG Type to another HG Type.

The mean r-values were 0.76, 0.73, 0.76, 0.78, and 0.77 for maBLUP; 0.57, 0.55, 0.56, 0.60, and 0.56 for cBLUP; 0.82, 0.79, 0.80, 0.81, and 0.82 for gBLUP; and 0.71, 0.71, 0.73, 0.78, and 0.75 for sBLUP in all.87176, r500, r1000, r5000, and r10000 SNP sets, respectively. The averaged r-values were 0.76, 0.57, 0.81, and 0.73 for maBLUP, cBLUP, gBLUP, and sBLUP, respectively (**Supplementary Table S17**). These results show that maBLUP, gBLUP, and sBLUP had high r-values with r ≥ 0.71, except for cBLUP with r ≥ 0.55. This indicates that all four models, except cBLUP, can be used for prediction to select SCN resistance based on phenotypic data from one HG Type to another one.

The correlation among the prediction values from the three HG Types, 2.5.7, 7, and 1.3.6.7, showed high r-values (**Supplementary Table S18**). In the maBLUP model, the lowest correlation was r = 0.69 between HG 7.prediction and HG 1.3.6.7.prediction in all.87176 SNP set, while the highest was r = 0.88 between HG 2.5.7.prediction and HG 7.prediction in r500 SNP set. In the cBLUP model, the lowest correlation was r = 0.55 between HG 7.prediction and HG 1.3.6.7.prediction in all.87176SNP SNP set, and the highest was r = 0.92 between HG 2.5.7.prediction and HG 1.3.6.7.prediction in r5000 SNP set. In the gBLUP model, all correlations were very high with r ≥ 0.81 between each pair in all five SNP sets. In the sBLUP model, the correlation was r = 0.69 between HG 7 and HG 1.3.6.7 in all five SNP sets, and r = 0.83 between HG 2.5.7 and HG 7, and between HG 2.5.7 and HG 1.3.6.7 (**Supplementary Table S18**), indicating that resistant FI to one HG Type can be predicted using the FI value in another HG Type through GS.

### GWAS-derived SNP marker

The two GWAS-derived SNP marker sets, either m20 or m71, exhibited highest r-values (**Supplementary Tables S11**-**S13**; **Supplementary Figures 12a, b**, **13a, b**, **14a, b**; **Figures 6**-**8**). For HG Type 2.5.7 resistance, m20 showed r-values ≥ 0.77 in BA, BB, BRR, BL, and gBLUP models, except for r = 0.64 in rrBLUP and 0.66 in cBLUP in all.panel; ≥ 0.76 in all models except r = 0.51 in rrBLUP in Q1; and ≥ 0.72 in all models except r = 0.45 in rrBLUP in Q2. M71 exhibited high r-values across all seven GP models with r ≥ 0.71 in all, Q1, and Q2, even ≥ 0.85 in BA, BB, BRR, BL, and gBLUP models in all and Q1 panels (**Supplementary Table S11**, **Supplementary Figures 12a, b**). Similar r-values were observed for resistance to HG Type 7 and 1.3.6.7, with even higher r-values with r ≥ 0.91 for resistance to HG Type 1.3.6.7, exceeding 0.90 in BA, BB, BRR, BL, and gBLUP in all panel (**Supplementary Tables S12**, **S13**, **Supplementary Figures 13a, b**, **14a, b**). These results indicate that GWAS-derived SNP markers can be effectively used to select resistance to either HG Type 7, 2.5.7, or 1.3.6.7 in common bean breeding through GS.

### Genetic prediction using difference genomic models

**Figure 12:**
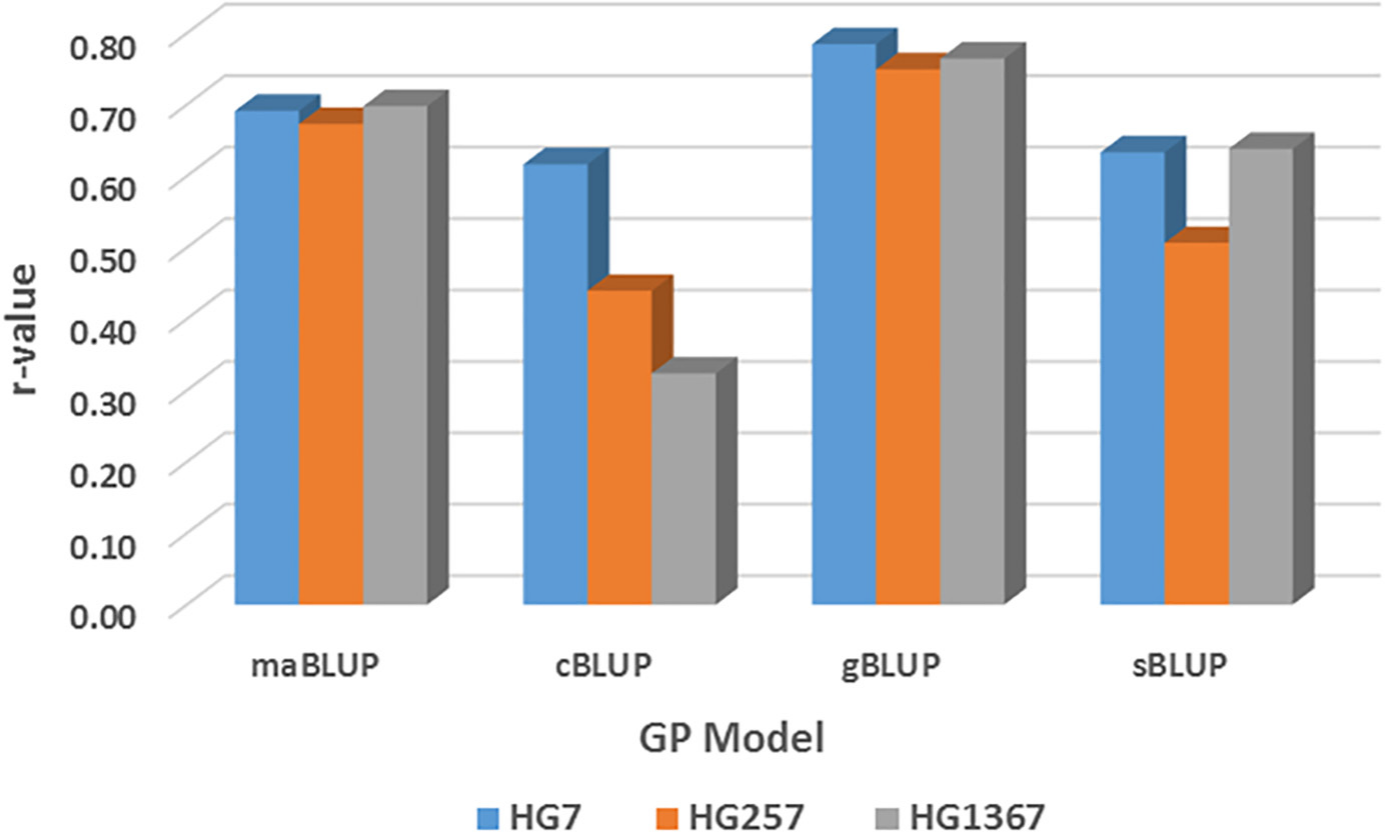
Genomic prediction (r-value) among four GP models, maBLUP, cBLUP, gBLUP, and sBLUP cross three SCN HG Types, HG 7 (race 6), HG 2.5.7 (race 5) and HG1.3.6.7 (race 14) using GAPIT 3 tool using randomly selected 10,000 SNPs comparisons, averaged r-value from the 11 GP pairs (combinations) of across- and cross-population.

Seven GP models (BA, BB, BRR, BL, rrBLUP, cBLUP, and gBLUP) were employed to estimate GP (r-values) for cross-population prediction (**Supplementary Tables S11**-**S13**, **Supplementary Figures 12a, b**, **13a, b**, **14a, b**; **Figures 6**-**8**); four models (maBLUP, cBLUP, gBLUP, and sBLUP) for both across- and cross-population prediction (**Supplementary Tables S16**, **S17**; **Figure 12**). Across all three panels (all, Q1, and Q2), all seven models demonstrated high r-values in each SNP set. BA, BB, BRR, BL, and gBLUP exhibited similar r-values, while rrBLUP and cBLUP showed slightly lower r-values (**Supplementary Tables S11**-**S13**). This suggests that the Bayesian models (BA, BB, BRR, or BL) and gBLUP are preferable for selecting SCN resistance in common bean through GS.

### Genomic heritability

In this study, genomic heritability (GH) was estimated using the rrBLUP model for resistance to the three SCN HG Types: 7, 2.5.7, and 1.3.6.7, across 10 different randomly selected SNP number sets ranging from 20 SNPs to 10,000 SNPs, in addition to two GWAS-derived SNP marker sets (20 and 71 markers - m20 and m71). This estimation was conducted through cross-prediction in three panels: all 354 common bean accessions, Q1 with 202 accessions, and Q2 with 152 accessions (**Supplementary Table S19**, **Supplementary Figure S16**).

The mean genomic heritability (GH) was 36.5%, 39.7%, and 41.4% in the all panel (354 accessions); 53.3%, 51.2%, and 59.8% in Q1 (202 accessions); and 48.7%, 51.0%, and 59.5% in Q2 (152 accessions) for resistance to HG Types 2.5.7, 7, and 1.3.6.7, respectively, averaged from the 12 different SNP number sets in cross-prediction. The results indicated that GH was highest for resistance to HG Type 1.3.6.7 in all three panels, second for HG Type 7 in all and Q2 panels, but second for HG Type 2.5.7 only in Q1 (**Supplementary Table S19**). This suggests that GH was highest for HG Type 1.3.6.7, followed by HG Type 7, and lowest for HG Type 2.5.7.

In most cases, as more SNPs were selected as a set, higher genomic heritability (GH) was observed for resistance to each HG Type in each panel (**Supplementary Table S19**). After reaching 500 SNPs, GH was consistently over 50% in most cases, suggesting that using 500 or more SNPs as a set for genomic selection for SCN resistance in common bean is feasible. The GH observed in the GWAS-derived marker sets, either m20 or m71, was slightly higher than that of the same SNP number sets, either r20 or r71, but lower than those sets in most cases when 500 SNPs or more were randomly selected (**Supplementary Table S19**).

### Genetic diversity and utilization of the SCN resistant germplasm accessions

**Figure 13:**
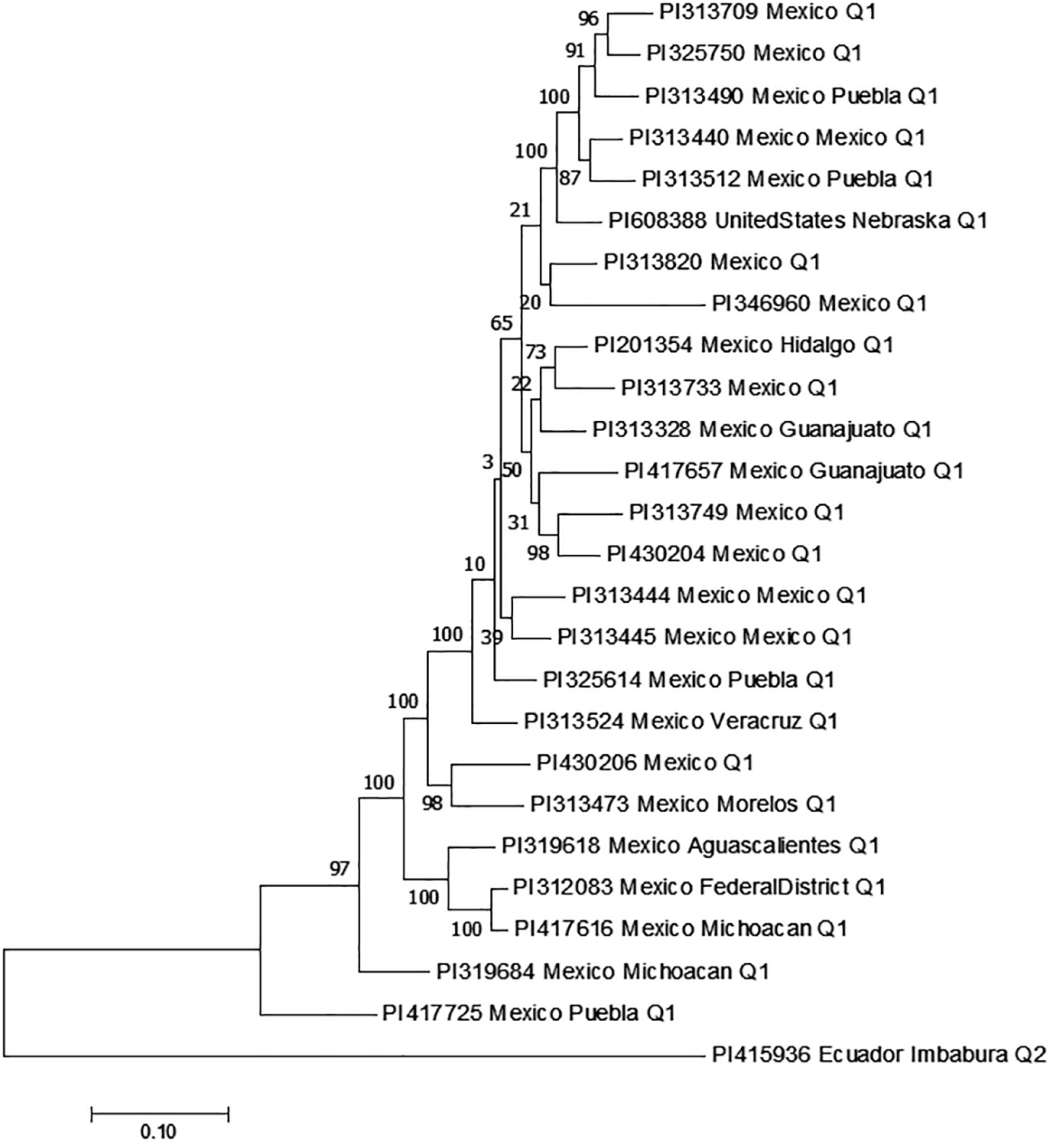
The phylogenetic tree created by the Maximum Likelihood (ML) method from MEGA 7 in 26 common bean germplasm accessions that were resistant to all three SCN HG Types 7, 2.5.7 and 1.3.6.7 with FI <10.

Among the 78 resistant accessions with a cyst nematode index (FI) of less than 10.0 for one or more of the three HG Types (7, 2.5.7, and 1.3.6.7) (**Supplementary Table S20**, **Supplementary Figure S17**), 26 accessions exhibited FI values below 10.0 for resistance to all three HG Types (**Table 6**, **Figure 13**). The remaining 52 accessions either had FI values above 10.0 for resistance to one of the three HG Types or had missing SCN phenotypic data (**Supplementary Table S20**).

Among the 26 accessions exhibiting an FI < 10 for all three SCN HG Types, 25 of them are present in Q1, with only PI 415936 from Ecuador appearing in Q2 (**Table 6**, **Figure 13**). Within Q1, 24 accessions originated from Mexico, while one accession from the United States. This suggests that the primary source of resistance to multiple HG Types is predominantly found in Mexico.

**Table 6:** Top 26 common bean accessions with SCN resistance FI <10.0 in three HG Types: HG Type 7 (race 6), HG Type 2.5.7 (race 5), and HG Type 1.3.6.7 (race 14).

Line_ID1	PI	Country	Q1	Q2	2Cluster	Race5 FI	Race6 FI	Race14 FI
PI313709_Mexico_Q1	PI313709	Mexico	1	0	Q1	1.8	7.1	1.2
PI313733_Mexico_Q1	PI313733	Mexico	1	0	Q1	1.1	2	1.5
PI313749_Mexico_Q1	PI313749	Mexico	1	0	Q1	3.3	9.9	2.1
PI313820_Mexico_Q1	PI313820	Mexico	0.998	0.002	Q1	2.4	5.5	2.1
PI325750_Mexico_Q1	PI325750	Mexico	1	0	Q1	1.8	2.9	3.5
PI346960_Mexico_Q1	PI346960	Mexico	1	0	Q1	3.7	3.5	3.5
PI430204_Mexico_Q1	PI430204	Mexico	1	0	Q1	4.9	5.5	2.1
PI430206_Mexico_Q1	PI430206	Mexico	0.998	0.002	Q1	0.9	9.4	3.9
PI319618_Mexico,Aguascalientes_Q1	PI319618	Mexico, Aguascalientes	0.999	0.001	Q1	4.6	7	3.3
PI312083_Mexico,FederalDistrict_Q1	PI312083	Mexico, FederalDistrict	1	0	Q1	4.4	7.7	7.7
PI313328_Mexico,Guanajuato_Q1	PI313328	Mexico,Guanajuato	1	0	Q1	3.3	7.5	1.6
PI417657_Mexico,Guanajuato_Q1	PI417657	Mexico,Guanajuato	1	0	Q1	4	3	2.8
PI201354_Mexico,Hidalgo_Q1	PI201354	Mexico,Hidalgo	1	0	Q1	1.6	4.7	2
PI313440_Mexico,Mexico_Q1	PI313440	Mexico,Mexico	1	0	Q1	2.5	6.6	4.1
PI313444_Mexico,Mexico_Q1	PI313444	Mexico,Mexico	1	0	Q1	1	2.7	2.3
PI313445_Mexico,Mexico_Q1	PI313445	Mexico,Mexico	1	0	Q1	2	2.1	2
PI319684_Mexico,Michoacan_Q1	PI319684	Mexico,Michoacan	0.974	0.026	Q1	5.8	5.3	1.6
PI417616_Mexico,Michoacan_Q1	PI417616	Mexico,Michoacan	1	0	Q1	5	6.3	2.2
PI313473_Mexico,Morelos_Q1	PI313473	Mexico,Morelos	1	0	Q1	3.8	8	2.8
PI313490_Mexico,Puebla_Q1	PI313490	Mexico,Puebla	1	0	Q1	3.1	5.1	2.4
PI313512_Mexico,Puebla_Q1	PI313512	Mexico,Puebla	1	0	Q1	9.1	3.5	4.6
PI325614_Mexico,Puebla_Q1	PI325614	Mexico,Puebla	1	0	Q1	4.1	9.1	1.5
PI417725_Mexico,Puebla_Q1	PI417725	Mexico,Puebla	0.922	0.078	Q1	9.9	4.7	3
PI313524_Mexico,Veracruz_Q1	PI313524	Mexico,Veracruz	1	0	Q1	1.5	2.6	1.1
PI608388_UnitedStates,Nebraska_Q1	PI608388	UnitedStates, Nebraska	0.999	0.001	Q1	2.4	5.8	1.7
PI415936_Ecuador,Imbabura_Q2	PI415936	Ecuador,Imbabura	0.002	0.998	Q2	4.3	5	2.1

The 78 accessions were sourced from 15 countries, with the majority comprising 50 accessions from Mexico. Additionally, there were 6 accessions from Ecuador and 4 from Peru, with 2 accessions each from Bolivia, Colombia, Costa Rica, Guatemala, India, and the United States. Furthermore, there was 1 accession each from Bulgaria, Canada, China, El Salvador, Japan, and Turkey (**Supplementary Table S20**). This distribution indicates that the majority of SCN-resistant lines originate from Mexico (64.1%), followed by the Ecuador-Peru region (12.8%), with the remaining countries contributing to the remainder (23.1%, with each country accounting for 0.6% or 1.3%). Analyzing the phylogenetic tree (**Supplementary Table S20**, **Supplementary Figure S17**), two main clusters are apparent. Specifically, 47 out of the 50 accessions from Mexico are grouped within cluster Q1. Meanwhile, the 6 accessions from Ecuador, 4 from Peru, 2 from Bolivia and Guatemala, and 1 from India, Bulgaria, and Mexico each form part of cluster Q2. Notably, two accessions, PI 417622 and PI 417624 from Mexico, appear as outliers. In Q2, four accessions—PI 201018 and PI 343950 from Guatemala, PI 535395 from Mexico, and PI 361321 from India—are somewhat distant from the main cluster. Consequently, they are identified as outliers within Q2.

## Discussion

### Genome-wide association study and SNP marker identification for SCN resistance

In this study, a total of 40 SNPs were identified to be associated with SCN resistance (**Supplementary Table S6**) in the all-panel of 354 common bean accessions using 87,176 SNPs distributed across 11 chromosomes (**Supplementary Figure S2**). These 40 SNP markers exhibited a LOD (-log(P-value)) greater than 6.24, which is the Bonferroni threshold value for the all-panel, as determined by at least one of the five models used, alongside a *t*-test for resistance to one of the three SCN HG Types (**Supplementary Table S6**).

Furthermore, 24 SNPs were identified in the Q1 panel of 202 accessions using 71,972 SNPs, with a LOD greater than 6.16, the Bonferroni threshold value for the Q1 panel, detected by at least one of the five models, alongside a *t*-test for resistance to one of the three HG Types (**Supplementary Table S7**). Similarly, 15 SNPs were identified in the Q2 panel of 152 accessions using 55,933 SNPs, with a LOD greater than 6.05, the Bonferroni threshold value for the Q2 panel, detected by at least one of the five models, alongside a *t*-test for resistance to one of the three HG Types (**Supplementary Table S8**).

Combining all SNP markers from the three panels for resistance to the three SCN HG Types, specific SNPs were selected as molecular markers for resistance to each SCN HG Type in each of the three panels based on their high LOD values across GWAS models. A total of 71 SNPs were reported, with 20 of them selected as markers associated with resistance to one or more SCN HG Types across different common bean panels. This led to the identification of nine QTL regions located on Chrs 2, 2, 3, 6, 6, 9, 10, 10, and 11, respectively (**Supplementary Tables S21a, b**). Notably, differences were observed based on domestication, with certain QTLs being identified predominantly in specific sub-populations. Furthermore, the QTLs exhibited varying resistance to different HG Types (races), highlighting the complexity of SCN resistance in common bean across different genetic backgrounds (**Supplementary Table S21**).

The 71 SNPs reported in this study represent a novel contribution to understanding SCN resistance in common bean. However, several SNP regions have been previously identified by studies including Jain et al. (2019); Shi et al. (2021), and Wen et al. (2019). **Supplementary Table S22** lists a total of 153 SNP markers, including the 71 from current study. Within this dataset, 17 SNP regions are found to have SNP markers reported within a <3 Mbp distance in previous studies. For instance, the SNP Chr02_30212013 identified for resistance to HG 1.3.6.7 in this study corresponds to SNP ss715639664_Chr02_30457681 reported by Jain et al. (2019) for resistance to HG Type 0, with a distance of 245,668 bp. This highlights the potential overlap and consistency in identifying genomic regions associated with SCN resistance across different studies using different SCN populations and HG Types.

### Genomic prediction for genomic selection of SCN resistance

Genomic selection (GS) has been extensively explored across numerous crops, including maize, rice, soybean, and wheat, for diverse agronomic traits and stress tolerances (Albrecht et al., 2011; Battenfield et al., 2016; Bernardo, 1996; Duhnen et al., 2017; Heffner et al., 2011; Jarquin et al., 2016; Onogi et al., 2016; Piepho, 2009; Poland et al., 2012; Rutkoski et al., 2011; Shikha et al., 2017; Spindel et al., 2015; Technow et al., 2013; Xavier et al., 2016; Zhang et al., 2017). The estimation of genomic breeding values is pivotal in GS, with various approaches proposed, including BLUP methods (rrBLUP, maBLUP, cBLUP, gBLUP, sBLUP), and Bayesian methods (Bayes A, Bayes B, Bayes LASSO (BL), and Bayes ridge regression (BRR)). Assessment of selection prediction accuracy is commonly performed using Pearson’s correlation coefficient (r) between the genomic estimated breeding values (GEBV) and observed values for each trait in the validation (testing) set across different models.

In this study, genomic prediction (GP) was conducted across three common bean panels: the all.panel comprising 354 accessions, the Q1 (Mesoamerican) panel with 202 accessions, and the Q2 (Andean) panel comprising 152 accessions. Seven GP models (BA, BB, BRR, BL, rrBLUP, cBLUP, and gBLUP) were employed for cross-population prediction, while five models (maBLUP, cBLUP, gBLUP, sBLUP, and rrBLUP) were utilized for both across- and cross-population predictions. GP performance, measured by the r-value, was assessed across various SNP sets and training/testing ratios (fold) in cross-prediction. Additionally, GP was evaluated in across-prediction scenarios among the three panels (all, Q1, and Q2).

GP estimation by different SNP set: The genomic prediction (GP) analysis was conducted using 12 SNP sets, comprising 10 different randomly selected SNP number sets and two sets derived from genome-wide association studies (GWAS) for resistance to the three SCN HG Types across three panels, employing seven GP models. Our findings revealed several key findings: Firstly, the correlation coefficient (r-value) increased with the inclusion of more SNPs in the SNP set, however, it had similar r-value when a SNP set had 500 or more SNPs, indicating a positive relationship between SNP number and prediction accuracy. Secondly, the r-value exhibited a similar trend across the three SCN HG Types, albeit with minor differences, suggesting consistent prediction accuracy regardless of HG Type. Thirdly, the r-value was consistently lower in Q2 compared to the all.panel and Q1, indicating potentially reduced prediction accuracy in this sub-population. Lastly, the r-value was notably lower when only 20 SNPs were randomly selected as a set (r20), indicating decreased prediction accuracy with a smaller number of SNPs. These findings align with previous studies by Shi et al. (2021) and Wen et al. (2019) on the similar trait of SCN resistance in common beans, as well as with other trait prediction analyses reported in the literature (Bhattarai et al., 2022, 2023; Ravelombola et al., 2021, 2019, 2020; Shi et al., 2021). Overall, our results contribute to understanding the factors influencing prediction accuracy in genomic selection and highlight the importance of SNP selection in enhancing prediction performance.
Training/testing ratio (fold): Training/testing ratio (fold): GP (r-value) was estimated in nine folds from 2-fold (training set: testing = 1: 1) in cross-prediction for the resistance to three SCN HG Types, 7 (HG 7; race 6), 2,5,7 (HG 2.5.7; race 5), and 1.3.6.7 (HG 1.3.6.7; race 14) in three panels: all 354 common bean accessions, Q1 - 202 accessions, and Q2 - 152 accessions estimated by rrBLUP (**Supplementary Table S14**, **Supplementary Figure S15**). All folds from 2 to 10 had similar r-value in each of three common bean panels, all, Q1, or Q2, but Q2 had smaller r-value than other two due less size of the Q2 panel, showing either the size of training set or testing set would affect the GA. These findings also align with previous studies (Bhattarai et al., 2022, 2023; Ravelombola et al., 2021, 2019, 2020; Shi et al., 2022, 2021; Wen et al., 2019).
Across population prediction: In this study, genomic prediction (GP) accuracy (r-value) was estimated using rrBLUP across different SNP number sets ranging from 20 to 10,000 SNPs. The prediction focused on across-population scenarios, either from the Mesoamerican (Q1; 202 accessions) to Andean (Q2; 152 accessions) common bean accessions, or vice versa, for resistance to three SCN HG Types: 2.5.7, 7, and 1.3.6.7 (**Supplementary Table S15**, **Figure 9**). The results revealed relatively low prediction accuracy in across-population prediction between the two subpopulations, suggesting distinct genetic backgrounds influencing SCN resistance. However, leveraging a mixed population as a training set demonstrated high prediction accuracy for either subpopulation, implying the potential of genomic selection (GS) for enhancing SCN resistance in common bean across diverse genetic backgrounds. In this study, negative PA was observed in some cases during across-population predictions from Q1 to Q2 or Q2 to Q1 (**Supplementary Table S15**), indicating challenges in accurately predicting SCN phenotypes. These results may underscore the need to refine models, incorporate additional markers, or account for environmental interactions to improve prediction accuracy. Despite the occurrence of negative PA, the GS framework remains valuable for identifying useful patterns to improve SCN tolerance.
GWAS-derived SNP markers as the SNP set: In this study, we also evaluated genomic prediction (GP) using 20 (m20) and 71 SNP markers (m71) derived from genome-wide association studies (GWAS). Both sets of markers showed higher prediction accuracy (r-values) for resistance to all three SCN HG Types compared to other SNP sets. However, the m71 GWAS-derived SNP markers exhibited the highest GP accuracy, indicating the presence of multiple alleles with minor effects contributing to SCN resistance in common bean. This approach, combining marker-assisted selection (MAS) and genomic selection (GS), can be valuable in real-world breeding programs, despite potential biases in predictive ability when using SNP markers from the same GWAS panel (Shi et al., 2021). Similar approaches have been successfully applied in predicting genetic architecture for various traits in different crops, including wheat, soybean, and others (Ali et al., 2020; Qin et al., 2019; Ravelombola et al., 2021, 2019, 2020; Spindel et al., 2016; Zhang et al., 2016b). Therefore, employing MAS and GS through genomic estimated breeding values (GEBVs) using associated SNP markers holds promise for molecular breeding aimed at enhancing SCN resistance in common bean, as well as for improving other quantitative traits in diverse plant species.
GP for different HG types: The genomic prediction (GP) analysis for SCN resistance across different HG Types (races) revealed several key findings. Despite variations in HG Types, the host common bean accessions did not exhibit distinct patterns that could distinguish between them, indicating a lack or little of differential host response to different SCN HG Types (races). High correlations were observed among the three races based on both phenotypic data and predicted genomic estimated breeding values (GEBV), suggesting a shared genetic basis for resistance across HG Types. Furthermore, the high genomic accuracy (GA) in predicting GEBV from one HG Type to another indicates the robustness of the resistance mechanisms against SCN evolution. Analysis of 78 resistant accessions (**Supplementary Table S20**) demonstrated that resistance to one HG Type often conferred resistance to others, with few instances of intermediate susceptibility. This supports the notion that SCN resistance may predate SCN pathogen stress and that *gene-for-gene co-evolution may not be necessary for SCN resistance in common beans*. Consistent with previous studies, accessions displayed varying degrees of resistance to different HG Types, with more robust resistance observed against HG123567 compared to HG 2.5.7. However, the correlation between HG 2.5.7 and HG123567 resistance, as reported by Wen et al. (2019), was relatively low (r=0.34), suggesting challenges in predicting GEBV between races based solely on SCN phenotypic data. These findings underscore the complexity of SCN resistance in common beans and highlight the need for further research to elucidate the underlying genetic mechanisms and optimize breeding strategies.

### Genetic diversity, domestication, utilization of SCN resistant resources

Based on principal component analysis (PCA) and phylogenetic analysis, the 354 accessions were categorized into two clusters: Q1 representing Mesoamerican and Q2 representing Andean domestication. Within Q1, further subdivision resulted in three groups (g1, g2, and g3), while Q2 split into two groups (p1 and p2), resulting in a total of five sub-populations: Q1g1, Q1g2, Q1g3, Q2p1, and Q2p2 (**Supplementary Table S1**). Among the 78 accessions that were resistant to one or more HG Types, 52 accessions were in Q1g1 predominantly from Mexico, with minor representation from Guatemala, Colombia, and the United States; eight accessions in Q1g2, evenly distributed between Costa Rica and Mexico, with additional accessions from Canada, Colombia, El Salvador, and the United States; five accessions in Q1g3 with two originating from India and one each from China, Japan, and Turkey; and 13 accessions in Q2p1 with the majority from Ecuador, followed by representation from Peru, Bolivia, and Bulgaria (**Supplementary Table S20**). Notably, Q2p2 did not contain any accessions displaying SCN resistance with FI < 20, except for PI 165617, which exhibited a FI of 19.4, indicating resistance to HG 1.3.6.7. However, PI 165617 showed higher FIs of 48.6 for HG 7 and 30.7 for HG 2.5.7 resistance (**Supplementary Table S1**).

The phylogenetic analysis of 354 common bean germplasm accessions revealed two distinct sub-populations, Q1 and Q2, delineated by domestication into Mesoamerican and Andean origins, respectively. This differentiation was evident when analyzing both 6,600 randomly selected SNPs (**Supplementary Figure S3-1**) and 20 SNP markers associated with resistance to SCN HG Types 7, 2.5.7, or 1.3.6.7 (**Supplementary Figure S18**). Notably, within each sub-population, resistant accessions tended to cluster together, highlighting the presence of two distinct types of SCN-resistant resources (**Supplementary Table S20**). Further analysis of the most highly associated 13 SNP markers within QTL regions among the 78 SCN-resistant accessions reaffirmed this pattern, illustrating the existence of two distinct SCN-resistant resource pools based on Mesoamerican Q1 and Andean Q2 domestications (**Supplementary Figure S19**). Evidences have been shown that common bean originated in the Mesoamerica, and Andean gene pool originated through different migration events from the Mesoamerican populations (Bitocchi et al., 2012). It is likely that SCN originated along with the major host soybean in northern China, and the nematode was introduced to America in 19th and/or 20th century (Riggs, 2004). These findings suggest that SCN resistance may predate SCN pathogen stress in common beans and that resistance can evolve either in wild ancestry or in domestication without the necessity of a gene-for-gene interaction between the plant host and pathogen (PagÃ¡n and GarcÃ­a-Arenal, 2018). The reason for why there were more SCN-resistant accessions in Mesoamerican gene pool than in Andean gene pool is unclear, but it is possible that the resistance evolved through the different domestication processes between the two regions. The identification of these two SCN-resistant resource pools offers valuable insights for breeders seeking to enhance SCN resistance in common bean breeding programs, providing guidance on parent selection strategies.

## Conclusion

In this study, we evaluated 354 USDA common bean germplasm accessions for resistance to SCN HG Types 7, 2.5.7, and 1.3.6.7 under controlled greenhouse conditions. Notably, 26 lines exhibited resistance to all three populations of different HG types, while 78 lines showed resistance to at least one HG type. We identified four QTL regions associated with resistance to each HG type, highlighting potential genetic targets for breeding programs. Our comprehensive genomic prediction (GP) analysis demonstrated the superior performance of Bayesian models (BA, BB, BRR, BL) and gBLUP in predicting SCN resistance. Despite observing low prediction accuracy (PA) across populations between Mesoamerican and Andean common bean accessions, using a mixed population as a training set showed high PA for predicting either sub-population. These findings underscore the potential of SNP markers for marker-assisted selection and genomic selection in common bean breeding programs, facilitating the identification of SCN-resistant lines and plants. Furthermore, we observed robust resistance mechanisms across the three HG types, with variations in highly associated SNP markers and QTL between domestications (Mesoamerican and Andean), suggesting pre-existing resistance to SCN. Our investigation into genomic heritability (GH) highlights the potential of larger SNP sets for reliable genomic selection in SCN resistance breeding programs. Additionally, genome-wide association study results identified specific SNP markers associated with SCN resistance across different bean panels and HG Types, offering valuable targets for marker-assisted selection and further genetic studies. Overall, our study significantly advances genomic selection strategies for enhancing SCN resistance in common bean, providing a promising approach to accelerate breeding efforts against this detrimental pest.

## Data Availability

The datasets presented in this study can be found in online repositories. The names of the repository/repositories and accession number(s) can be found in the article/**Supplementary Material**.
